# Electronic Symptom Reporting Between Patient and Provider for Improved Health Care Service Quality: A Systematic Review of Randomized Controlled Trials. Part 1: State of the Art

**DOI:** 10.2196/jmir.2214

**Published:** 2012-10-03

**Authors:** Monika Alise Johansen, Eva Henriksen, Alexander Horsch, Tibor Schuster, Gro K Rosvold Berntsen

**Affiliations:** ^1^Norwegian Centre for Integrated Care and TelemedicineUniversity Hospital of North NorwayTromsøNorway; ^2^Institute of Medical Statistics and EpidemiologyTechnische Universität MünchenMunichGermany; ^3^Department of Telemedicine and e-HealthInstitute of Clinical MedicineUniversity of TromsøTromsøNorway

**Keywords:** Electronic symptom reporting, physician-patient relationship, patient participation, shared decision making, review, consultation, monitoring, self-management

## Abstract

**Background:**

Over the last two decades, the number of studies on electronic symptom reporting has increased greatly. However, the field is very heterogeneous: the choices of patient groups, health service innovations, and research targets seem to involve a broad range of foci. To move the field forward, it is necessary to build on work that has been done and direct further research to the areas holding most promise. Therefore, we conducted a comprehensive review of randomized controlled trials (RCTs) focusing on electronic communication between patient and provider to improve health care service quality, presented in two parts. Part 2 investigates the methodological quality and effects of the RCTs, and demonstrates some promising benefits of electronic symptom reporting.

**Objective:**

To give a comprehensive overview of the most mature part of this emerging field regarding (1) patient groups, (2) health service innovations, and (3) research targets relevant to electronic symptom reporting.

**Methods:**

We searched Medline, EMBASE, PsycINFO, Cochrane Central Register of Controlled Trials, and IEEE Xplore for original studies presented in English-language articles published from 1990 to November 2011. Inclusion criteria were RCTs of interventions where patients or parents reported health information electronically to the health care system for health care purposes and were given feedback.

**Results:**

Of 642 records identified, we included 32 articles representing 29 studies. The included articles were published from 2002, with 24 published during the last 5 years. The following five patient groups were represented: respiratory and lung diseases (12 studies), cancer (6), psychiatry (6), cardiovascular (3), and diabetes (1). In addition to these, 1 study had a mix of three groups. All included studies, except 1, focused on long-term conditions. We identified four categories of health service innovations: consultation support (7 studies), monitoring with clinician support (12), self-management with clinician support (9), and therapy (1). Most of the research (21/29, 72%) was conducted within four combinations: consultation support innovation in the cancer group (5/29, 17%), monitoring innovation in the respiratory and lung diseases group (8/29, 28%), and self-management innovations in psychiatry (4/29, 14%) and in the respiratory and lung diseases group (4/29, 14%). Research targets in the consultation support studies focused on increased patient centeredness, while monitoring and self-management mainly aimed at documenting health benefits. All except 1 study aiming for reduced health care costs were in the monitoring group.

**Conclusion:**

RCT-based research on electronic symptom reporting has developed enormously since 2002. Research including additional patient groups or new combinations of patient groups with the four identified health service innovations can be expected in the near future. We suggest that developing a generic model (not diagnosis specific) for electronic patient symptom reporting for long-term conditions may benefit the field.

## Introduction

This paper presents the first part of a comprehensive review of randomized controlled trials (RCTs) focusing on electronic communication between patient and provider to improve health care service quality. Part 1 presents an overview of patient groups, health service innovations, and research targets relevant to electronic symptom reporting. Part 2 examines the methodological quality of the RCTs and summarizes effects and benefits of electronic symptom reporting of the methodologically best RCT studies from the reported data [1].

Patients today, including the elderly and less-educated [2], are quite motivated to use electronic services [3-5]. A new approach is being taken in countries with high e-readiness [6], focusing on the patient-provider partnership and information technology to promote patient-centered health care [7,8] and shared decision making [9,10]. In this approach, a new concept to improve patient centeredness is emerging, reflected in the rapidly rising number of studies during the past few years [11]: patients or parents reporting symptoms or health information electronically [11]. The patient reports to health care personnel, an institution, or a system, where the receiver processes and interprets the data and provides feedback to the patient [11]. The purpose, in general, is improved health care service quality, for example, by improving or avoiding consultation [11].

Patients support the idea of previsit reporting electronically [12-15] and believe it will improve the level of care and effectiveness [13,14]. Wald et al reported that when 2027 primary care patients, who already had an account to the secure electronic health record-connected Internet patient portal, were invited to provide health information electronically before consultation, 70% actually did so [16]. Patients felt more prepared for the visit and that their provider had more accurate information about them [16]. On the health system level, trials of electronic symptom reporting suggest that it may be possible to substitute about one-third or more of face-to-face consultations in primary care settings [17,18]. Further examples of the benefits that patients, health care personnel, and the health care system can gain from these tools are provided in part 2 of this study [1].

### Patient Groups, Research Targets, and Health Service Innovations

A preliminary review conducted in 2010, based on abstracts, found that most studies in the field were small in terms of number of patients involved and are best described as feasibility studies [11]. This also called attention to the impression that electronic symptom reporting seemed to be more relevant for some patient groups or health conditions, such as complex conditions where it is challenging to cover all relevant issues during one short visit [11]. Examples are cancer [19], asthma [20,21], congestive heart failure [22,23], pain [24], neurological disorders [25], and mental health issues [3,26,27]. On the other hand, electronic symptom reporting was also used for less-severe problems such as atopic eczema [28], for follow-up after surgery [11,29,30], and in general primary care settings [17,18].

However, the health service innovations and research targets seem to involve a broad range of foci with regard to choices of patient groups, technology, organizational implementation, and outcome measures [11]. In such a heterogeneous field it is difficult to assess which patient groups are most likely to benefit, which types of interventions are the most promising, and which outcomes are likely to be improved by the interventions. This is not surprising, since telemedicine and eHealth are complex systems representing a blend of many disciplines [31]. To move the field forward it is necessary to create a map of what has been examined so far and to encourage more research into the areas holding most promise and the areas that are still unknowns on the map. No systematic review has yet addressed this theme, to the best of our knowledge.

### Objective

The overall aim of the review was to systematically assemble the knowledge focusing on electronic communication between patient and provider to improve health care service quality. We wanted to limit our work to the most mature stage of a complex intervention before taking a service into ordinary use, the RCTs [32,33].

The objective for this first part of the review was to create a comprehensive overview of the most mature part of the field and to clarify what has been investigated so far with regard to different patient groups, health service innovations, and research targets relevant to electronic symptom reporting. Patient groups refers to either health conditions or to health services in cases where the trial did not focus on a specific diagnosis.

## Methods

The review in general followed the Preferred Reporting Items for Systematic Reviews and Meta-analyses (PRISMA) recommendations [34]. To further improve the quality, we consulted the Cochrane handbook [35] for data extraction. The group conducting the review has a multidisciplinary background, including experience in medical and epidemiological research (GB, AH, TS), RCT methodology and statistics (TS, GB, AH), telemedicine and medical informatics (MAJ, EH, AH, TS), theoretical knowledge of electronic symptom reporting (MAJ, EH), and experience from earlier review work (AH, GB, TS).

### Inclusion and Exclusion Criteria

Studies had to meet the following inclusion criteria: (1) it had to be an original study, (2) patients or parents in the intervention group had to report symptoms or health information electronically, either to clinical health care personnel or to a system, where the receiver processed and interpreted the data for health care purposes and provided feedback (we accepted that the feedback did not have to be given electronically; the focus was on asynchronous systems that can be established within the health care system, including e-diaries and personal health records accessible to health care providers), (3) the information reported had to be about the patient symptoms and health status at the time of reporting or during the preceding few days, and (4) it had to be an RCT comparing electronic symptom reporting versus a control group where symptom or health information was not received by the health care professionals or systems. This means that the control group may have varied from standard care or waiting lists to control groups where patients reported their symptoms or health information electronically but where this health information was not received by the health care professionals or the interpreting systems.

Studies fulfilling one or more of the following criteria were excluded: (1) retrospective questionnaires, prevalence surveys, general screening on the Internet, and tests of medications, (2) electronic communication requiring the patient and health care personnel to be present simultaneously, for instance in a video conference or through instant messaging, (3) automatic biometric measurements, since these are defined as reporting of signs, not symptoms, and 4) voice diary.

### Search Methods for Identification of Studies

We searched the following electronic literature databases: Medline, EMBASE, PsycINFO, the Cochrane Central Register of Controlled Trials, and IEEE Xplore. The search was limited to publications from 1990 (due to no knowledge of older publication within this field), human medicine, English language, and RCTs (for PsycINFO: Treatment Outcome/Randomized Clinical Trial). We restricted EMBASE searches to exclude records imported from Medline. The first search was conducted in May 2011, and the search was last updated in October and November 2011.

We reviewed known eligible publications to identify possible indexing terms and relevant search words. It was necessary to establish a comprehensive search for two reasons. First, this is a new area without any established terms defining the field. Second, medical and medical informatics expressions evolve over time, where new terms appear and traditional terms are replaced by more specific ones [36]. Scope, indexing, and thesaurus terms are not equivalent in each database [37]. Thus, we had to adapt the initial Medline search to the search in other databases, keeping them as close as possible to the initial search.

We accessed Medline, EMBASE, and PsycINFO through the Ovid interface. Cochrane and the Ovid searches were built around four search files (What, Who, Why, and How), with a logical OR within the files, and an AND between the files. The Medline search was based on medical subject headings (MeSH) and the Text Words (TW) field to search titles and abstract information. The What file consisted of 22 search terms, including 3 MeSH terms, for symptoms and synonyms, such as “health data” or “health information*”. The Who file searched for “patient*” and “parent*” plus 16 relevant MeSH terms. The Why file included 51 search terms, of which only 3 were MeSH terms, for “self-report*”, “pre-report*”, and synonyms. Finally, the How file contained 38 search terms, including 11 MeSH terms, for the possible technologies involved. The search strategies were pilot tested and modified several times to ensure that they identified eligible publications. The Medline search strategy and search terms can be found in Multimedia Appendix 1.

The IEEE Xplore search had to be constructed in a different way because the limitation to a maximum of 10 search terms and 6 wildcards made it impossible to reuse the advanced Ovid searches. Since IEEE Xplore in general included few RCT metadata, we conducted a search for “RCT* OR (randomi* AND control* AND trial*)”.

We did not include articles based on hand searches of reference lists, due to the Cochrane warning that “positive studies are more likely to be cited” and that “retrieving literature by scanning reference lists may thus produce a biased sample of studies” [35] (Cochrane 10.2.2.3, Citation bias). The only exception was if an article classified as relevant was a secondary analysis of an RCT, in which case we included the article presenting the primary analysis from the reference list.

### Data Collection and Analysis

#### Selection of Studies

**Figure 1:**
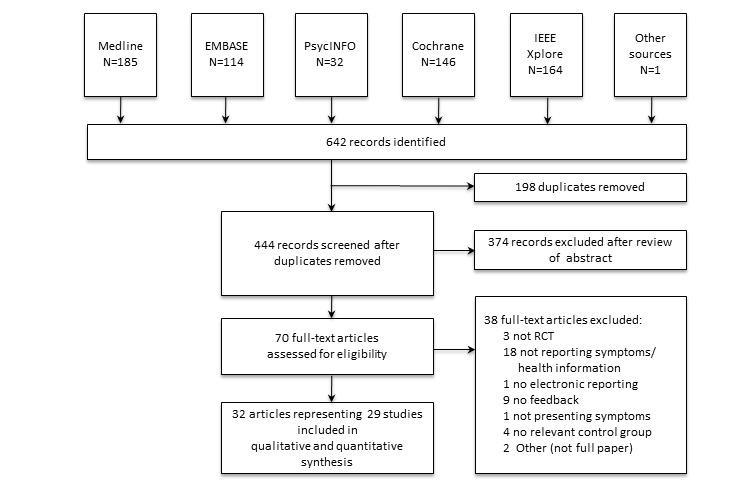
Process for searching and selecting randomized controlled trials (RCTs) of electronic symptom reporting. The study flow diagram distinguishes between records and studies. A record is a source providing information about a study, presenting at a minimum an article title and abstract. Studies are the overall research projects themselves (here the RCTs), which may be represented by more than 1 article.

Search results were exported to EndNote X3 (Thomson Reuters, Carlsbad, CA, USA) for merging of databases, identification and deletion of duplicates, and review management. Abstract and full-text review were conducted independently, as presented in Figure 1, by two authors (MAJ and EH), who extracted data based on the inclusion and exclusion criteria into a structured spreadsheet. In the abstract review, we used only the information available in one specific abstract, and in the full-text review only the information available in one specific article, to determine eligibility for inclusion. All disagreements were resolved by consensus discussions. In a few cases, one author (GB) was consulted for full-text review and involved in the final conclusion.

#### Combining Articles

Sometimes authors reported primary and secondary analyses from the same RCT in 2 separate articles. Other authors conducted a small RCT pilot before the main RCT. In these cases, we allowed both articles, if we judged both to be relevant, to be separately included and evaluated in the review. However, we counted and present them as 1 RCT study and 2 articles.

We linked articles deemed not to be relevant, but that published design, methodological, or theoretical information for an included study, to the included article when we extracted data and when presenting the characteristics of each study.

#### Data Extraction and Management

From each included article, authors MAJ or EH extracted variables, guided by the Cochrane data collection checklist [35] (Table 7.3.a in the Cochrane handbook), in addition to study-specific variables. These in total 84 variables represent (1) eligibility criteria, (2) study design and duration, (3) assessment of methodological quality including evaluation of the risk of bias in the results, (4) patient groups (either health conditions or health services in cases where the trial did not focus on specific diagnoses), (5) health services interventions and the corresponding control group, and (6) outcome measures and results relevant to electronic symptom reporting. A full presentation of the extracted variables and the citations can be found on the website of the Norwegian Centre for Integrated Care and Telemedicine [38].

#### Patient Groups, Health Service Innovations, and Research Targets

Due to the heterogeneity and complexity of the studies regarding patient groups, health service innovations, and research targets, and to provide a richer source of evidence, we chose an approach combining quantitative and qualitative narrative evaluation of the selected articles [39]. Qualitative methods are useful for exploring key domains in health service research [40]. The data were explored using content analysis to break them down into categories (or typologies) relevant to this review [41]. Under the qualitative analysis, we treated the text of individual studies more as a whole to identify major themes and categories, and then compared and contrasted them with those of the other studies [39].

The resulting patient groups depended on whether we found articles focusing on health services types without focusing on specific diagnoses. If we found only articles focusing on specific diagnoses, we defined the resulting patient groups by their health condition and categorized them as in our preliminary review [11], by the use of International Classification of Primary Care (ICPC) [42].

The research targets were classified according to the six areas of health service quality defined by the Institute of Medicine (IOM), which state that health care should be safe, effective in terms of health benefits (mortality, morbidity, and quality of life), patient centered, timely, efficient, and equitable [43]. Outcomes were in addition categorized according to who benefited: patients, health professionals, or the health care system. Table 1 shows the cross-link between who benefits, general clinical outcomes and the more specific outcomes variables extracted for this review, as well as the IOM outcomes.

**Table 1:** Research target typology: extracted outcomes grouped by who benefits from the intervention and Institute of Medicine (IOM) quality areas for health care [43]

Outcomes benefitting	Extracted outcome variables	IOM quality area
Patients	Clinical outcomes Improved health care service for patient Resource utilization for patient Satisfaction for patient Other benefits and results Unintended effects	Patient centeredness Health benefits Safety Timeliness for receiver
Health care professionals	Improved health care service for doctors and others Resource utilization for doctors and others Satisfaction for doctors and others Other benefits and results Unintended effects	Efficiency: resource utilization (for health professionals) Timeliness for health professionals
Health care system	Avoided consultations Other benefits and results Unintended effects	Efficiency: health care costs Efficiency: duration or time Equitability

## Results

### Selection of Studies

Of 642 records identified through the search and 444 abstracts reviewed, 32 articles and 29 studies were included (Figure 1) [44-75]. Three studies were reported in 2 articles, where 2 studies reported primary and secondary analyses in 2 separate articles ([59,60] and [73,74]), and 1 study had conducted a small RCT pilot before the main RCT [52,53].

The full-text review resulted in agreement on 49 articles, while we discussed 21 articles to reach consensus. Of these, 7 were finally included and 14 were excluded. The third author was involved in the discussion of 9 articles, where 3 were finally included and 6 excluded. Classification of abstracts from the database searches as not relevant or potentially relevant can be found in Multimedia Appendix 2.

### Background Data

The 32 articles were published over 10 years, from 2002 to November 2011, with most (n = 24) published in the last 5 years. All 29 studies, except 2, were conducted in Western countries: 12 in the United States, 4 in the United Kingdom, 3 in the Netherlands, 2 in Sweden, 2 in Switzerland, and 1 each in Australia, Denmark, Norway, Singapore, Spain, and Taiwan. Four of the parallel studies had three arms [48,57,69,71]; all the others had two. All except 2 studies randomly allocated patients; the exceptions used cluster randomization: 1 randomized primary care practices [73,74], and 1 randomized clinics [49]. All studies included both genders with an average of 60% females (ranges 37.5%-93%). In nearly two-thirds (20/32) of the articles, the first author is female.

### Patient Groups

We found no articles that did not focus on specific diagnoses. Thus, resulting patient groups were all defined by their health condition and categorized as in our preliminary review [11], mainly by the use of ICPC [42]. The exception is cancer, which is not a separate category in ICPC but is represented in a large and distinct body of the literature and is therefore presented separately.

The included articles resulted in five specific patient groups and one mixed group. Of the total of 29 studies, respiratory and lung diseases are clearly the largest group, with 12 studies (see Table 2).

All of the included studies, except that of Yardley et al [66], focused on long-term conditions or issues.

### Health Service Innovations

In the content analysis of the included studies, we identified the following four categories of health service innovations: (1) consultation support, (2) monitoring with clinician support, (3) self-management with clinician support, and (4) therapy.

*Consultation support *includes patients or parents reporting symptoms or health information electronically prior to a consultation, where the main focus is how this affects the consultation. *Monitoring with clinician support *includes patients following a monitoring program. The patient reports measurements and health data, and a health care professional monitors the patient’s disease or treatment. *Self-management with clinician support *might include some monitoring elements, but most important is that patients in these studies have to follow a self-management program, with communication and supporting feedback from clinicians provided to the innovation group. Self-management programs focus on problem-solving skills to overcome barriers, making action plans and carrying them out, and education to increase patients’ confidence and their ability to manage their symptoms and illness [76,77]. The fourth category, *therapy*, is different from all the other categories in that it comprises innovations where the whole treatment, and all communication between therapists and patients, is conducted exclusively electronically. No specific self-management program or module is included in the therapy category.

We categorized studies that were difficult to categorize because they included elements from both monitoring and self-management according to our interpretation of the studies’ main purpose. The following studies were categorized as monitoring but included some self-management elements: Chan et al [52,53], Jan et al [55], Rasmussen et al [57], Guendelman et al [54], and Nguyen et al 2009 [61]. On the other hand, Nguyen et al [68] and van der Meer et al [67] were categorized as self-management, but included some monitoring elements.

Table 2 presents the resulting health service innovations according to the resulting patient groups. The monitoring category is largest, including 12 studies, while self-management includes 9 studies, consultation 7, and therapy 1.

Most of the consultation support innovations were conducted in the cancer patient group (5/7), and most of the monitoring studies were in the respiratory and lung diseases group (8/12). In self-management, nearly half (4/9) of the studies were conducted in the field of psychiatry or in the respiratory and lung diseases patient group (4/9).

**Table 2:** Reviewed randomized controlled trials of electronic symptom reporting, by health service innovation category and patient group^a^.

Patient group	Consultation support studies	Monitoring with clinician support studies	Self-management with clinician support studies	Therapy study	Total studies
Cancer	5 studies: Berry et al [44]; Boyes et al [45]; Ruland et al [46]; Ruland et al [47]; Velikova et al [48]	1 study: Kearney et al [51]	0	0	6
Respiratory and lung diseases: asthma	0	6 studies in 7 articles: Willems et al [58]; Chan et al [53] and Chan et al [52]; Guendelman et al [54]; Jan et al [55]; Prabhakaran et al [56]; Rasmussen et al [57]	1 study: van der Meer et al [67]	0	7
Respiratory and lung diseases: chronic obstructive pulmonary disease	0	2 studies in 3 articles: Lewis et al [59] (health care use) and Lewis et al [60] (quality of life); Nguyen et al [61]	1 study: Nguyen et al [68]	0	3
Respiratory and lung diseases: other	0	0	2 studies: DeVito Dabbs [65]; Yardley et al [66]	0	2
Cardiovascular diseases	0	3 studies: Carrasco et al [62]; Santamore et al [63]; Schwarz et al [64]	0	0	3
Psychiatry	1 study: Stevens et al [49]	0	4 studies: Berger et al [69]; Bergström et al [70]; Vernmark et al [71]; Oerlemans et al [72]	1 study: Wagner et al [75]	6
Diabetes	0	0	1 study in 2 articles: Williams et al [74] and Glasgow et al [73]	0	1
Mixed	1 study: Leveille et al [50]	0	0	0	1
Total studies	7	12	9	1	29

^a ^Articles were identified in a comprehensive search in Medline, EMBASE, PsycINFO, Cochrane Central Register of Controlled Trials, and IEEE Xplore from 1990 to November 2011, and were published in the time period 2002–2011. References with *and *between them are articles that belong to the same study.

Table 3 presents the location of the patient at the time of communication and who the patient’s main communication partner was [44-75,78-80]. Combining the results from Table 2 and Table 3 gives an overview of the main communication partner and the physical context of the patient’s reporting situation within the different health innovation areas.

In the group of consultation support articles, symptom reporting was conducted at the clinic (one exception), and the physician, or both a physician and a nurse, was the main communication partner for the patient. In all the monitoring, self-management, and therapy articles, the patient was at home when reporting. The main communication partner was the nurse in monitoring studies (7 studies). In psychiatry self-management, psychologists were the main communication partners. A total of 3 self-management and 3 monitoring studies mainly used computer-tailored feedback to the patients, 5 with and 1 without nurse or physician support.

**Table 3:** Topic of reviewed randomized controlled trials of electronic symptom reporting, by patient’s location at time of symptom reporting and main communication partner^a^.

Main communication partner	Location of patient	
	Inside health care institution	Outside health care institution (at home)
Unclear	0	1 study: lung diseases [65]^b^
Physician at hospital	3 studies: cancer [45] + [48]; psychiatry [49]	1 study: cardiovascular [63]
Physician and nurse	3 studies: cancer [44,78]^c ^+ [46] + [47]	0
General practitioner or primary care physician	0	1 study: cardiovascular [62]
Psychologist	0	5 studies: psychiatry [75] + [69]^d ^+ [70] + [71]^d ^+ [72]
Nurse	0	9 studies: cancer [51,79]^c^; asthma [58,80]^c ^+ [52,53] + [56]; chronic obstructive pulmonary disease [59,60] + [61] + [68]; cardiovascular [64]; mixed [50]
CTF^e ^only	0	1 study: lung diseases [66]
CTF and physician	0	2 studies: asthma [55] + [57]
CTF and nurse	0	3 studies: asthma [67] + [54]; diabetes [73,74]

^a ^Articles were identified in a comprehensive search in Medline, EMBASE, PsycINFO, Cochrane Central Register of Controlled Trials, and IEEE Xplore from 1990 to November 2011, and were published in the time period 2002–2011.

^b ^Patients communicated with a transplant team, a transplant provider, a coordinator, and a transplant coordinator at the hospital. The professions of these actors are not clearly defined [65].

^c ^Articles 78, 79, and 80 were deemed not to be relevant, but included information necessary to understand the study in question.

^d ^Conducted mainly by students [69,71] under the supervision of a more experienced or senior psychologist.

^e ^Computer-tailored feedback.

### Characteristics of Included Studies in Relation to Health Innovation Categories

The included studies are presented according to the resulting health service innovation categories (Table 4, Table 5, Table 6, and Table 7). The tables describe methods, participants and relevant considerations and components for replicating the intervention, according to Cochrane’s minimum requirements [35] (11.2.2). In addition, the main findings column presents the results of individual studies, as recommended by PRISMA [34]. Since the studies are heterogeneous with respect to disease, interventions, and outcomes, the summary descriptions are not easily standardized. Thus, we produced a more detailed and comprehensive table than is common in most reviews.

The 7 consultation studies involved more patients per study than did the monitoring and self-management support studies: 2342 patients (range 52–878, median 241). The 12 monitoring studies included a total of 1824 patients (range 17–321, median 120). The 9 self-management studies included 2242 patients (range 50–886, median 88), and the therapy study included 55 patients; 10 studies included fewer than 100 patients.

Consultation studies generally followed patients through one consultation only, while the duration of other interventions varied from 1 to 12 months, where more than half lasted 4 months or less, 6 lasted between 6 and 8 months, and only 4 lasted as long as 12 months.

**Table 4:** Summary description of studies on consultation support in the reviewed randomized controlled trials of electronic symptom reporting^a^.

Patient group	Trial and country^b^	Participant characteristics^c^	Study characteristics^d^	Health service innovation: consultation support	Main findings and research targets^e^
**Cancer**					
	Berry et al [44]; (Wolpin et al [78]); USA	262 clinicians from 2 clinics; 660 cancer patients, 18–86 (mean 54) years; female % not reported	Design: P + 2; inside clinic Duration: 2 visits (before treatment and 4–6 weeks later)	Enhancing patient–provider communication with electronic self-report assessment for cancer *Intervention: ESRA-C* ^f^: a color graphical summary of the participant’s self-reported symptoms and quality-of-life issues with predetermined thresholds flagged was printed and handed to the clinician immediately before the targeted clinic visit. No recommendations offered. *Control*: ESRA-C questionnaires were filled, but no summary was handed to the clinician.	*Berry et al [44]: Primary*: The likelihood of symptoms and quality-of-life issues being discussed between clinicians and patients differed by randomized group and depended on whether a symptoms and quality-of-life issue was first reported as problematic (*P *= .032). *Secondary: *Clinic visits were similar with regard to duration between groups, and clinicians reported the summary as useful. *Wolpin et al [78]*: The ESRA-C was easy for patients to use and acceptable across a range of user characteristics. *Research targets: *Patient centeredness, duration, resource utilization
	Boyes et al [45]; Australia	80 cancer patients, 20–85 years (mean not reported); female 59.5%	Design: P + 2; inside clinic Duration: before visit, 4 times	Effect of giving oncologist a summary of the cancer patient’s self-reported psychosocial well-being *Intervention: *Touch-screen survey filled out before oncologist visit. Computer scored the answers and a printed summary report was placed in the patient’s file for consideration during consultation. Suggested strategies for managing identified issues were included. *Control: *Touch-screen survey filled out, but no results made available to oncologist.	*Unclear primary outcome. *Intervention patients who reported a debilitating physical symptom at visit 2 were significantly less likely to report a debilitating physical symptom at visit 3 compared with control patients (odds ratio 2.8, *P *= .04). Reductions in levels of anxiety, depression, and perceived needs among intervention patients were not significantly different from those among control patients. *Research targets: *Health benefits, patient centeredness
	Ruland et al [46]; USA	14 physicians, 14 nurse practitioners; 52 cancer patients; 23–77 (mean 56.3) years; female 59%	Design: P + 2; inside clinic Duration: 1 consultation	Supporting shared decision making *All patients *scheduled for an outpatient visit used the system on a tablet computer to report their symptoms and preferences prior to consultation. The system highlighted for clinicians what symptoms patients were experiencing, including severity, degree of bother, and importance to patients. This information was printed and provided to the patient and clinician in the *experimental group *but not in the *control group*.	*Primary: *Significantly greater congruence between patients’ reported symptoms and those addressed by their clinicians in the experimental group. *Secondary: *The system scored high on ease of use. There were no significant group differences in patient satisfaction. *Research target: *Patient centeredness
	Ruland et al [47]; Norway	145 cancer patients (leukemia or lymphoma); ≥18 (mean in intervention: 50, in control: 49) years; female 38%	Design: P + 2; inside clinic Duration: up to 1 year (once per encounter during treatment, once per week during hospital stay, once per outpatient visit in up to 4 visits)	Effects of a computer-supported interactive tailored patient assessment tool Both groups used *Choice, an interactive tailored patient assessment*, touchpad tablet PC, for symptom assessments prior to inpatient and outpatient visits. The assessment summary, which displayed the patient’s self-reported symptoms, problems, and distress in rank order of the patient’s need for support, was provided to physicians and nurses in the *intervention group*. *Control group *patients used exactly the same tool, but the clinicians were not given any information from the patient’s assessment.	*Primary: *Significantly more symptoms were addressed in the intervention group patient charts than in those of the control group. *Secondary: *Symptom distress in the intervention group decreased significantly over time in 11 (58%) of 19 symptom/problem categories versus 2 (10%) for the control group. Need for symptom management support over time also decreased significantly more for the intervention group than the control group in 13 (68%) symptom categories. *Research targets: *Patient centeredness, health benefits, resource utilization
	Velikova et al [48]; UK	28 physicians, 286 oncology patients; age range not reported; mean age 54.9 years; female 73%	Design: P + 3; inside clinic Duration: approximately 6 months	Improving communication and patient well-being *Intervention group *completed touch-screen HRQL^g ^questionnaires in the waiting room before every encounter. A summary was presented to physicians. *Attention-control group *also completed HRQL questionnaires on touch-screen computer, but summary was not presented to physicians. *Control group *used no touch-screen measurement of HRQL before clinic encounters.	*Several primary outcomes: *Intervention and attention-control groups had better HRQL than the control group (*P *= .006, *P *= .01, respectively), but the intervention and attention-control groups were not significantly different (*P *= .80). A positive effect on emotional well-being was associated with data feedback (*P *= .008) but not with instrument completion (*P *= .12). A larger proportion of intervention patients showed clinically meaningful improvement in HRQL. More frequent discussion of chronic nonspecific symptoms (*P *= .03) was found in the intervention group, without prolonging encounters. The clinicians found the information useful. There was no detectable effect on patient management (*P *= .60). In the intervention patients, HRQL improvement was associated with explicit use of HRQL data (*P *= .016), discussion of pain, and role function (*P *= .046). *Research targets: *Health benefits, patient centeredness, duration, resource utilization
**Psychiatry**	Stevens et al [49]; USA	878 potential behavioral concern patients from 9 clinics; 11–20 (mean 13.9) years; female 54%	Design: C + 2; inside clinic Duration: 1 consultation	Does screening increase clinicians’ recognition of behavior concerns? The *Health eTouch system *collected self-report data from patients in the waiting rooms. At 5 sites, patients’ screening results were printed and given to the primary care provider just before the face-to-face encounter (*immediate-results condition*). At 4 sites, the 1-page summary was mailed to the primary care provider 2–3 business days later (*delayed-results condition*). Providers had immediate access to screening results for youth reporting thoughts about suicide, regardless of group assignment.	*Primary: *In intent-to-treat analysis, difference approached but did not reach statistical significance (*P *= .058). However, if all youths who endorsed suicidal ideation, regardless of original condition assignment, were included in the immediate-results condition, then 68% of youths in the immediate-results condition who screened positive were identified as having a problem by their pediatrician, which was significantly higher than the recognition rate of 52% in the delayed-results condition (*P *= .001). *Research targets: *Health benefits, patient centeredness
**Mixed**	Leveille et al [50]; (Allen et al [81]); USA	34 physicians, 241 patients (depression, chronic pain, and mobility difficulty); 22–86 years (mean not reported); female 57%	Design: P+ 2; outside, probably at home Duration: from 4 weeks until index visit (start unclear).	Nurse coaching to promote patient–primary care physician discussion *Intervention: PatientSite *was used to enhance patient–provider communication regarding 3 common conditions (chronic pain, depression, and impaired mobility) during upcoming visits. Delivered online by nurse e-coaches, the intervention involved a standardized set of emails and worksheets targeting self-efficacy, patient education, and motivation to improve health. *Control: *Patients received a general message through PatientSite containing URL links to US government websites with general health information.	*Several primary outcomes: *Detection and treatment of the target conditions (1-week postvisit survey) and symptom burden related to these conditions. Similar high percentages of intervention (85%) and control (80%) participants reported discussing their condition during their primary care physician visit. More intervention than control patients reported their primary care physician gave them specific advice about their health (94% vs 84%; *P *= .03) and referred them to a specialist (51% vs 28%; *P *= .002). Intervention participants reported somewhat higher satisfaction than controls (*P *= .07). Results showed no significant differences in detection or management of screened conditions, symptom ratings, and quality of life between groups. *Research targets: *Health benefits, patient centeredness

^a ^Articles were identified in a comprehensive search in Medline, EMBASE, PsycINFO, Cochrane Central Register of Controlled Trials, and IEEE Xplore from 1990 to November 2011, and were published in the time period 2002–2011.

^b ^Main author, main reference. References in parentheses contributed relevant study information on the study in question.

^c ^Number of clinicians, number of patients with diagnosis, age range (mean) of patients, percentage female patients.

^d ^Design (P = parallel group design, CO = crossover, C = cluster, F = factorial, O = other, U = unclear; + number of study arms), where symptom reporting took place (outside or in the home; or inside a clinic), and duration of intervention.

^e ^Main findings are in general presented as in the original article and refer to primary outcome if clearly defined and secondary outcomes considered relevant for the scope of the study. Research targets refers to the six areas of health service quality defined by the Institute of Medicine [43].

^f ^Electronic Self-Report Assessment-Cancer.

^g ^Health-related quality of life.

**Table 5:** Summary description of studies on monitoring in the reviewed randomized controlled trials of electronic symptom reporting^a^.

Patient group	Trial and country^b^	Participant characteristics^c^	Study characteristics^d^	Health service innovation: monitoring	Main findings and research targets^e^
**Cancer**					
	Kearney et al [51]; (McCann et al [79]); UK	112 breast, lung, or colorectal cancer patients; >18 (mean 56) years; female 76.8%	Design: P + 2; outside/home Duration: 4 cycles of chemotherapy (12–16 weeks)	Management of chemotherapy-related toxicity *Intervention*: A mobile phone system (ASyMS) was used in the morning, evening, and at any time patients felt unwell on days 1–14 following their first 4 cycles of chemotherapy. Patients completed an electronic symptom questionnaire on their mobile, including reporting their temperature. Patients immediately received written feedback on the phone. Clinicians were advised to contact patients within 1 hour after receipt of a red alert. The system’s alert to physician was based on a risk model. *Control: *Received standard care.	*Unclear primary outcome. *2 of the 6 symptoms measured (fatigue and hand–foot syndrome) showed statistical significance between the 2 randomized groups: higher reports of fatigue in the control group and lower reports of hand–foot syndrome in the control group. *Research target: *Health benefits
**Respiratory and lung disease**					
	Chan et al [53]; and the small prestudy Chan et al [52]; USA	120 children with persistent asthma; 6-17 (mean in intervention: 10.2, in control: 9.0) years; female 37.5% (Chan et al [52]: 10 children)	Design: P + 2; outside/home Duration: 12 months	Internet-based monitoring and education of children with asthma *Intervention: The Asthma In-Home Monitoring *group received 3 in-person visits and Internet-based education. They reported asthma signs and symptoms daily. Peak flow videos were sent twice per week for 6 weeks and then once weekly. Case manager scored results based on standardized checklists. *Control: *Traditional in-person education and case management over 6 scheduled visits. *Both groups: *The case manager contacted patients by email (intervention) or telephone (control) twice per week for 6 weeks and once per week thereafter to review their information. The patients were able to contact the case manager by email (intervention) or telephone (control) whenever needed. Asthma education in both groups followed the same curriculum.	*Unclear primary outcome (both studies). *Virtual patients had higher metered-dose inhaler with valved holding chamber technique scores than did the office-based group at 52 weeks (94% vs 89%), had greater adherence to daily asthma symptom diary submission (35.4% vs 20.8%), had less participant time (636 vs 713 patient-months), and were older. Caregivers in both groups perceived an increase in quality of life and an increase in asthma knowledge scores from baseline. There were no other differences in therapeutic or disease control outcome measures. *Research targets: *Health benefits, patient centeredness, resource utilization
	Guendelman et al [54]; USA	134 children with asthma; 8–16 (mean in intervention: 12, in control: 12.2) years; female in intervention: 60%; in control: 63%	Design: P + 2; outside/home Duration: 3 months	Asthma outcomes and self-management behaviors *Intervention: Health Buddy *enabled children to assess and monitor their asthma symptoms and quality of life daily and to transmit this information to a nurse. A protocol based on clinical practice guidelines consisting of 10 questions was designed. Patients answered daily queries from a nurse by pressing 1 of 4 buttons. Patients received immediate feedback from the Health Buddy. Asthma facts and trivia questions, which changed daily, were included to pique children’s curiosity and enhance learning. *Control: *Participants used a standard asthma diary.	*Primary: *After adjusting for covariates, the odds of having any limitation in activity were significantly (*P *= .03) lower for Health Buddy children *Secondary: *The intervention group was also significantly (*P *= .01) less likely to report peak flow readings in the yellow or red zone or to make urgent calls to the hospital (*P *= .05). Self-care behaviors also improved far more for the intervention group. *Research targets: *Health benefits, patient centeredness
	Jan et al [55]; Taiwan	164 children with persistent asthma; 6–12 (mean in intervention: 10.9, in control: 9.9) years; female in intervention: 60.3%, in control: 63.2%	Design: P + 2; outside/home Duration: 3 months	Interactive asthma monitoring *Intervention: *With *Blue Angel for Asthma Kids*, children completed the electronic asthma diary and recorded symptoms, need for rescue medication, and PEF^f ^values, preferably daily. The tool comprised a 3-color real-time warning system accompanied by a treatment plan. Patients were asked to follow instructions given by the computer and the physician; thereafter, the decision support system was used to check whether asthma had been brought under control. Physicians then instructed the patients by email or telephone to increase, decrease, or continue the usual treatment. (See Rasmussen et al [57] for a comparable intervention.) *Control: *Patients recorded the same PEF values and asthma symptoms on paper, and received the same optimal clinical care, education program (as part of usual care), and support from asthma management teams. Their written asthma diary was supplemented by instructions for self-management.	*Unclear primary outcome.* When the 2 groups were compared with regard to change from baseline, the children in the intervention group had a significant decrease of nighttime (*P *= .028) and daytime symptoms (*P *= .009) compared with the children in the control group. The adherence rates of therapeutic and diagnostic monitoring, global assessment of asthma control, knowledge of asthma self-management, and quality of life of caregivers were all significantly higher in the intervention group than in the conventional asthma care group. *Research targets: *Health benefits, patient centeredness
	Prabhakaran et al [56]; Singapore	120 asthma patients; mean age in intervention: 37, in control: 40 years); female in intervention: 65%, in control: 53%	Design: P + 2; outside/home Duration: 3 months	Asthma monitoring *Intervention: *Patients received text messages to assist with asthma management, daily for 2 weeks, thereafter weekly for 10 weeks. New data were compared with previous results, and the frequency of reliever use was analyzed by the server receiving the data. If the value was too high (preset threshold) or the patient did not reply to 3 consecutive messages, an alert email was sent to the asthma nurse. All alerts were verified by the asthma nurse through telephone contact with the patients. *Control: *Patients had no text messaging support.	*Unclear primary outcome. *Asthma-control test scores improved for 36 participants in the intervention group compared with 28 in the control group (*P *= .113). Number of nebulizations decreased in 54 participants in the control group compared with 50 in the intervention group (*P *= .053). Emergency room visits decreased in 57 participants in the control group compared with 51 in the intervention group (*P *= .063). Admission rates did not decrease in either group (*P *= .5). The mean response rate to the messages was 82%, and 92% in the intervention group were satisfied with the text messaging service. *Research targets: *Health benefits, health care costs, patient centeredness
	Rasmussen et al [57]; Denmark	300 asthma patients; 18–45 (mean 29.5) years; female 69%	Design: P + 3; outside/home Duration: 6 months	Asthma monitoring *Intervention 1: Internet-based monitoring. *Patients completed an electronic diary and recorded symptoms, need for rescue medication, and PEF values, preferably daily. The Internet action plan calculated the level of asthma control and offered the patient advice on what to do next by using a 3-color warning system (green, yellow, and red). If the patient scored red, an email was sent to the physician. The physician used a decision support system to decide the level of treatment. Physician instructed patients by email or telephone. (See Jan et al [55] for a comparable intervention.) *Intervention 2: Specialist monitoring group *were taught how to use a peak flow meter and a written action plan daily (comprising a 3-color warning system based on the symptom score and PEF values) to adjust their medication. *Control: *In the *GP* ^g ^ *group *the GP assessed the patient’s asthma symptoms and test results and from this decided the patient’s need for pharmaceutical treatment. The patients in the GP group did not receive any treatment or information about asthma from the study physician.	*Several primary outcomes: *Treatment and monitoring with the Internet-based management tool led to more significant improvement in the Internet group than in the other 2 groups regarding *asthma symptoms *(Internet vs specialist: odds ratio2.64, *P *= .002; Internet vs GP: odds ratio 3.26; *P *< .001), *quality of life *(Internet vs specialist: odds ratio 2.21, *P *= .03; Internet vs GP: odds ratio 2.10, *P *= .04), *lung function *(Internet vs specialist: odds ratio 3.26, *P *= .002; Internet vs GP: odds ratio 4.86, *P *< .001), and *airway responsiveness *(Internet) vs GP: odds ratio 3.06, *P *= .02). *Research target: *Health benefits
	Willems et al [58]; (Willems et al [80]); the Netherlands	109 patients with mild to moderate asthma; 56 children 7–18 (mean 11) years, 53 adults ≥18 (mean 46) years; female 55.6%	Design: P + 2; outside/home Duration: 12 months	Nurse-led telemonitoring *Intervention: *Lung function values and symptoms registered at home twice daily on a portable handheld device (diaries) were transferred to the asthma nurse (main caregiver) monthly or when having asthma complaints. The nurse studied the data daily and classified the asthma following a stepwise intervention protocol. The nurse was allowed to decrease (after 3 months of stable asthma) or increase asthma medication by 1 step. Physician was consulted only if necessary. *Control: *Regular care.	*Willems et al [58]: Primary: *No significantly improved asthma-specific quality of life; *secondary*: no significant decrease of asthma symptoms or medical consumption (time and medication). *Willems et al [80]: *Higher mean health care costs per patient in the intervention group. A decrease in the price of the asthma monitor will substantially increase the probability of the program being cost effective. *Research targets: *Health benefits, resource utilization, health care costs
	Lewis et al [59] (Reduce health care use) and Lewis et al [60] (quality of life); UK	40 patients with moderate to severe COPD^h ^who had completed at least 12 sessions of outpatient pulmonary rehabilitation; mean age in [59] in intervention: 67, in control: 70 years; mean age in [60] for intervention: 70, for control: 73 years; female 50%	Design: P + 2; outside/home Duration: 6 months + 6-month follow-up	Home telemonitors to reduce health care use [59], and improve quality of life for patients [60] *Intervention: *Patients received standard care plus *Docobo HealthHUB *handheld monitors at home for 26 weeks followed by 26 weeks of standard care (for observation and follow-up). During the monitoring period, patients recorded their symptoms and physical observations twice daily. Data were transmitted automatically at night via the home telephone line. Nurses could access the data through a website and receive alerting email messages if certain conditions were detected. *Control: *Standard care for 1 year.	*Reduced health care use: Primary: *No significant differences between the groups in hospital admissions [59]; *Secondary: *No significant differences between the groups in emergency room visits, days in hospital, or contacts with the specialist COPD community nurse team during the monitoring period, but fewer primary care contacts for chest problems (*P *< .03) in the intervention group [59]. After the monitors were removed, no significant differences were found between the groups for any of the health care contacts (*P *> .20 throughout) [59]. *Quality of life: Primary: *No significant differences in quality-of-life scores between the groups at any time, or consistently within either group over time [60]. *Research targets: *Health care costs, health benefits
	Nguyen et al [61]; USA	17 patients with moderate to severe and stable COPD; mean 68 (SD 11) years; female 65%	Design: P + 2; outside/home Duration: 6 months	A cell phone-based exercise persistence intervention postrehabilitation for COPD *All participants *developed an individualized exercise plan, were issued a pedometer and exercise booklet, and were trained to log their daily exercise and symptoms. *Intervention: MOBILE-coached *patients submitted symptoms and exercise daily, and received immediate summary feedback from server and weekly reinforcement feedback by text message from nurse; reports of worsening symptoms were automatically flagged to the nurse for follow-up. *Control: MOBILE self-monitored: *Entered and submitted the same information on the cell phone, but no (information and) alarm to nurse and no coaching feedback from nurse.	*Unclear primary outcome. *Small feasibility study. Logging exercise and symptoms was easy, and keeping track of their exercise helped patients remain active. There were no significant differences between groups over time in maximal workload, 6-minute walk distance, or health-related quality of life (*P *> .05); however, MOBILE-self-monitored increased total steps per day, whereas MOBILE-coached logged fewer steps over 6 months (*P *= .04). *Research targets: *Health benefits, patient centeredness
**Cardiovascular disease**					
	Carrasco et al [62]; Spain	38 GPs, 285 hypertensive patients; (age range not reported), mean 62 years; female 40%	Design: P + 2; outside/home Duration: 6 months	Text message-based Patient–GP interaction on control of hypertension *Intervention: *Patients sent the mean results of blood pressure self-monitoring 4 times a week, and heart rate and body weight once a week. They could complete an optional questionnaire during each wireless application protocol session. GPs accessed the data sent via the Web and could send a text message regarding any related issue to the patient’s phone. *Control: *Followed the same protocol, except that they recorded the data on paper and could only deliver it to their GP personally at the routine visits.	*Primary: *The influence of the interaction between patient and GP, in a nonspecialized setting, in the selected type of hypertensive patients, did not significantly improve the degree of hypertension control; *Secondary: *the course of hypertension during follow-up, adherence to the protocol, results of quality-of-life and anxiety questionnaires, or economic aspects such as the number of consultations or hospital admissions did not significantly improve. *Research targets: *Patient centeredness, health benefits, health care costs
	Santamore et al [63]; USA	321 cardiovascular disease patients; 18–85 (mean in intervention: 62, in control: 63.2) years; female % not reported	Design: P + 2; outside/home Duration: 8 months	Telemedicine System to Decrease Cardiovascular Disease Risk *All patients *received a manometer with memory and a pedometer. *Intervention: *Patients exchanged data with their care provider via the Internet. Patient could enter data daily, or for several days at once. Patients reported weight, blood pressure and heart rate, physical activity (steps/day), and cigarette smoking, and received feedback on cardiovascular disease risk reduction. Data for 10–15 patients were presented simultaneously on the provider screen. Out-of-limits parameter (red) values were presented first. *Control group *received usual care plus manometer and pedometer.	*Unclear primary outcome. *Systolic and diastolic blood pressures decreased significantly in both groups. The decreases in systolic blood pressure were greater in the intervention group (*P *< .05). For both groups, low-density lipoprotein decreased and high-density lipoprotein remained unchanged. In diabetic patients, blood glucose and glycated hemoglobin decreased significantly (*P *< .01) only in the intervention group. In nondiabetic patients, the risk of diabetes and metabolic syndrome score decreased (*P *< .01) only in the intervention group. Rates of usage of the telemedicine system were very high (92%). This rate of self-monitoring greatly exceeded the self-monitoring rate in controls (48%). The telemedicine-entered blood pressure values were similar to the meter-recorded values and to the office values. *Research targets: *Health benefits, patient centeredness
	Schwarz et al [64]; USA	102 heart failure patients; 65–94 (mean 78.1) years; female 52%	Design: P + 2; outside/home Duration: 3 months	Telemonitoring of heart failure patients and their caregivers *Intervention: e-Cardiocom electronic home monitoring *system measured weight daily. The device asked the participants to answer yes or no to questions about symptoms. The heart failure care manager, an advanced practice nurse, was responsible for daily monitoring of parameters. Measurements outside of prescribed parameters were automatically displayed, resulting in the nurse calling the caregiver in the dyad to further assess the situation, provide education, and update the medication regimen. The nurse notified the primary physician or cardiologist about the patient’s status as needed. *Control group: *Usual care.	*Several primary outcomes *(reducing subsequent hospital readmission, emergency department visits, and cost; and increasing the time between discharge and readmission). There were no significant differences due to telemonitoring for any outcomes. *Research targets: *Health care costs

^a ^Articles were identified in a comprehensive search in Medline, EMBASE, PsycINFO, Cochrane Central Register of Controlled Trials, and IEEE Xplore from 1990 to November 2011, and were published in the time period 2002–2011.

^b ^Main author, main reference. References with *and *between them are articles that belong to the same study. References in brackets contributed relevant study information on the study in question.

^c ^Number of clinicians, number of patients with diagnosis, age range (mean) of patients, percentage female patients.

^d ^Design (P = parallel group design, CO = crossover, C = cluster, F = factorial, O = other, U = unclear; + number of study arms), where symptom reporting took place (outside or in the home; or inside a clinic), and duration of intervention.

^e ^Main findings are in general presented as in the original article and refer to primary outcome if clearly defined and secondary outcomes considered relevant for the scope of the study. Research targets refers to the six areas of health service quality defined by the Institute of Medicine [43].

^f ^Peak expiratory flow rate.

^g ^General practitioner.

^h ^Chronic obstructive pulmonary disease.

**Table 6:** Summary description of studies on self-management in the reviewed randomized controlled trials of electronic symptom reporting^a^.

Patient group	Trial and country^b^	Participant characteristics^c^	Study characteristics^d^	Health service innovation: self-management	Main findings and research targets^e^
**Respiratory and lung disease**					
	DeVito Dabbs et al [65]; USA	34 lung transplant recipients; >18 (mean 56) years; female 40%	Design: P + 2; outside/home Duration: the first 2 months after discharge	Early self-care behaviors and follow-up after lung transplant *Intervention: Pocket Personal Assistant for Tracking Health *(Pocket PATH): In addition to standard care, patients used a handheld device to record health data, review data trends by using the screens and graphs, and follow feedback instructions regarding reporting changes to their transplant coordinator. *Control: *Used standard paper-and-pencil logs to record data. *Both groups *were instructed to contact their transplant coordinator for any clinical questions or issues. Follow-up was identical.	*Several primary outcomes: *Patients in the Pocket PATH group showed significantly higher ratings of self-care agency, performed self-care behaviors at significantly higher rates, and reported significantly better health-related quality of life than standard-care controls. *Research targets: *Health benefits, patient centeredness
	Yardley et al [66]; UK	714 participants with minor respiratory symptoms; 18–79 years (62.1% were <25); female 72.3%	Design: P + 2; outside/home Duration: 1 access + follow-up after 48 hours (332) and 4 weeks (214)	Web-based intervention providing tailored advice for self-management of minor respiratory symptoms *Intervention: *Web-based *Internet Doctor*-provided tailored computer-generated advice for minor respiratory symptoms (cough, sore throat, fever, and runny or stuffy nose). Participants could access 3 main pages: (1) diagnostic pages asking a series of questions about symptoms, and a complex algorithm providing appropriate tailored advice on whether they needed to contact health services (+ options to possible diagnoses), (2) treatment pages providing information about medication for symptoms, (3) Common Questions section addressing common concerns and misconceptions about symptoms and treatment. *Control group* got access to a static webpage providing the best existing patient information.	*Several primary outcomes: *1 month later the Internet Doctor resulted in higher levels of enablement (median 3 and 2, respectively; *P *= .03), and 11.6% (11) of participants consulted their doctor or used other health care services (mainly NHS Direct) for their symptoms, compared with a substantially greater proportion (21, 17.6%) in the control group (*P *= .22). *Secondary: *The Internet Doctor resulted in higher levels of satisfaction than the control information (mean 6.58 and 5.86, respectively; *P *= .002). Understanding of illness improved in the 48 hours following use of the Internet Doctor webpages, whereas it did not improve in the control group (mean change from baseline 0.21 and –0.06, respectively; *P *= .05). *Research targets: *Patient centeredness, resource utilization (for health professionals)
	van der Meer et al [67]; the Netherlands	200 asthma patients from 37 general practices and 1 academic outpatient department; 18–50 (mean in intervention: 36, in control: 37) years; females 69.5%	Design: P + 2; outside/home Duration: 12 months	Internet-based self-management plus education compared with usual care The *Internet-based self-management *program included weekly asthma-control monitoring and treatment advice, online and group education (face-to-face), and Web communication with a specialized asthma nurse, as an adjunct to usual care. Patients completed an electronic questionnaire on the website weekly and instantly received automated feedback on the state of their asthma control, along with advice on how to adjust their treatment (increasing or decreasing; contact asthma nurse). *Control group: *Usual physician-provided care according to Dutch general practice guidelines.	*Primary: *Asthma-related quality-of-life improvement of 0.5 point or more occurred in 54% and 27% of Internet and usual care patients, respectively (adjusted relative risk 2.00, confidence interval 1.38–3.04). Statistically significant, but not clinically significant. *Secondary: *Asthma control improved more in the Internet group than in the usual care group (adjusted difference –0.47, confidence interval –0.64 to –0.30). At 12 months, 63% of Internet patients and 52% of usual care patients reported symptom-free days in the previous 2 weeks (adjusted absolute difference 10.9%, confidence interval 0.05%–21.3%). *Research target: *Health benefits
	Nguyen et al [68]; USA	50 patients with moderate to severe chronic obstructive pulmonary disease; mean 69.5 years, range ± 8.5; female 44%	Design: P + 2; outside/home Duration: 6 months	Dyspnea self-management *Intervention*: Internet-based *(eDSMP) *dyspnea self-management *Control: *Face-to-face dyspnea self-management *(fDSMP)*. The content of the 2 programs was similar, focusing on education, skills training, and ongoing support for dyspnea self-management, including independent exercise. eDSMP participants submitted symptom and exercise information in real time via the PDA^f ^or website. fDSMP paper diaries were mailed weekly. Nurse-provided feedback via email (eDSMP) or telephone (fDSMP), weekly for the first month and then biweekly for the next 5 months. Contacts were expected to be as similar as possible for the 2 groups, except that automated email alerts were sent to the study nurses if worsening of symptoms or reports of not performing exercise for at least 3 consecutive days.	*Primary: *Both groups showed similar clinically meaningful changes in dyspnea with activities of daily living after 3 months and sustained these improvements at 6 months. *Secondary: *Self-reported endurance exercise time (*P *= .001), physical functioning (*P *= .04), and self-efficacy for managing dyspnea (*P *= .02) also showed positive improvements over time in both groups with no significant differences with respect to program modality. *Research targets: *Health benefits, patient centeredness
**Psychiatry**					
	Berger et al [69]; Switzerland	81 patients with social phobia; 19–62 (mean 37.2) years; female 53.1%	Design: P + 3; outside/home Duration: 10-week treatment + 6-month follow-up	Internet-based treatments of social phobia *Intervention 1: Guided Internet-based self-help *program with weekly scheduled email feedback by a therapist and the possibility to ask questions via email (response time maximum 3 days). *Intervention 2: Step-up on demand *(same as control) but with the possibility to step up to guidance by email (intervention 1) or telephone. Both groups used an online diary to report anxiety-provoking situations, and related thoughts, feelings, and behaviors. *Control: Pure self-help *program (by Internet) without any therapist support.	Significant symptom reductions in all 3 treatment groups with large effect sizes for *primary measures *(self-reported measures of symptoms of social phobia) and for *secondary outcome *measures (symptoms of depression, interpersonal problems, and general symptomatology). No significant between-group effects were found. No significant difference between the 3 conditions regarding diagnosis-free status, clinical change, dropout rates, or adherence measures such as lessons or exercises completed. High level of patient satisfaction overall, with a significant difference favoring the guided Internet-based self-help group. *Research targets: *Health benefits, patient centeredness
	Bergstrom et al [70]; Sweden	113 patients with panic disorder; >18 (mean in intervention: 33.8, in control: 34.6) years; female 61.5%	Design: P + 2; outside/home Duration: 10 weeks + 6-month follow-up	Internet-based CBT^g ^for patients with panic disorder *Intervention: *10 Web-based self-help modules, 1 per week, with information, exercises, and homework assignments, based on established CBT principles. Psychologist provided feedback, gave access to next module, and replied to other messages within 24 hours on regular weekdays. Only email contact. *Control: *Regular psychiatric care setting. Psychologists presented the same self-help program as above during weekly 2-hour sessions, supported by handouts. Homework assignments were addressed during group sessions.	*Primary: *Internet CBT is as effective as the more widely used group CBT. No significant between-group effects were found. *Secondary: *Internet treatment had superior cost-effectiveness ratios in relation to group treatment at both posttreatment and follow-up. *Research targets: *Health benefits, health care costs
	Vernmark et al [71]; Sweden	88 patients with major depression; age range not reported (mean 37); female 68%	Design: P + 3; outside/home Duration: 8 weeks (we don’t report from the 6-month follow-up, since the control group had received guided self-help by this time)	Internet-administered guided self-help versus individualized email therapy versus waiting lists *Intervention 1: Guided self-help *included weekly modules and homework assignments. Standard CBT components were presented. Therapists contributed with positive reinforcement on the progress made by participants. *Intervention 2: email therapy *did not use the self-help texts; all emails were written for the unique patient. The therapists had more or less the same role as in face-to-face psychological treatment. The contents of the email therapy overlapped with the self-help material but were tailored to each participant’s needs. The treatment was based on a protocol manual developed by the team. The third group was a *waiting-list control group*. Each therapist was responsible for 5 participants in each group.	*Primary: *Both the email therapy and the self-help groups improved in symptom reduction compared with the waiting-list condition (*P *= .002 and *P *= .06). The 2 treatments did not differ (*P *= .41). At posttreatment 34.5% of the guided self-help group, 30% of the email therapy group, and 13.8% of the waiting-list group reached the criteria of high-end state functioning (*P *= .17), (Beck Depression Inventory score <9). *Research target: *Health benefits
	Oerlemans et al [72]; the Netherlands	76 irritable bowel syndrome patients; age range not reported; mean in intervention: 35.9, for control: 40.6 years; female in intervention: 91.9%, in control: 76.9%	Design: P + 2; outside/mobile Duration: 4 weeks + 3-month follow-up	Intervening on cognitions and behavior in irritable bowel syndrome *Intervention: Patients *received standard care supplemented with a 4-week CBT e-intervention on PDAs. Patients completed 3 diaries daily. The data were immediately accessible to the psychologist, who during weeks 2–4 sent situational feedback based on CBT via text message. Feedback was standardized through a developed protocol. *Control: *Received standard care consisting of reassurance, dietary advice, and education from their general practitioner.	*Several primary outcomes: *No significant differences between groups for dysfunctional cognitions. Between-group comparisons after 4 weeks showed more overall quality-of-life improvement, more improvement in catastrophizing thoughts, and more pain improvement in the intervention group. Only improvement in catastrophizing thoughts persisted in the long term. The eHealth intervention seems feasible, since all intervention group patients completed the diaries 3 times a day for 4 weeks. *Research target: *Health benefits
**Diabetes**	Glasgow et al [73] and Williams et al [74]; USA	52 primary care physicians, 886 type 2 diabetes patients; >25 (mean in intervention: 61.48, in control: 64.63) years; female in intervention: 52.3%, in control: 50% ([73])	Design: C + 2; inside clinic Duration: 12 months	Interactive computer technology to assist patients and clinicians in emphasizing patient-centered communication and improved quality of care *Computer-assisted intervention *patients were asked to come 30 minutes early to 2 diabetes-related visits scheduled 6 months apart to complete a touch-screen assessment procedure and set self-management goals, received computer- tailored feedback and individualized action plans, received a printout on general health risks, met with a care manager, and received follow-up phone calls from care manager (nurse or medical assistant). Physician and care manager received printout of patient’s needs, self-management goals, and areas the patient wished to discuss. *Control patients *also completed touch-screen assessment procedure, received printout on general health risks, but did not set self-management goals, meet with a care manager, or receive follow-up phone calls.	*Glasgow et al [73]: Primary: *Significantly improved both the laboratory assays and patient-centered aspects of diabetes care that patients received compared with those in the control condition. *Secondary: *Overall improvement in lipids, glycated hemoglobin, quality of life, and depression scores; between-condition differences were not significant. *Williams et al [74]: Unclear primary outcome. *The intervention increased patient perception of autonomy support relative to a computer-based control condition (*P *< .05). Change in perceived competence partially mediated the effects of increased autonomy support on the change in lipids, diabetes distress, and depressive symptoms. The construct of autonomy support was separate from that of patient satisfaction. *Research targets: *Health benefits, patient centeredness

^a ^Articles were identified in a comprehensive search in Medline, EMBASE, PsycINFO, Cochrane Central Register of Controlled Trials, and IEEE Xplore from 1990 to November 2011, and were published in the time period 2002–2011.

^b ^Main author, main reference. References with *and *between them are articles that belong to the same study.

^c ^Number of clinicians, number of patients with diagnosis, age range (mean) of patients, percentage female patients.

^d ^Design (P = parallel group design, CO = crossover, C = cluster, F = factorial, O = other, U = unclear; + number of study arms), where symptom reporting took place (outside or in the home; or inside a clinic), and duration of intervention.

^e ^Main findings are in general presented as in the original article and refer to primary outcome if clearly defined and secondary outcomes considered relevant for the scope of the study. Research targets refers to the six areas of health service quality defined by the Institute of Medicine [43].

^f ^Personal digital assistant.

^g ^Cognitive behavioral therapy.

**Table 7:** Summary description of study on therapy in the reviewed randomized controlled trials of electronic symptom reporting^a^

Trial and country^b^	Participant characteristics^c^	Study characteristics^d^	Health service innovation: therapy	Main findings and research targets^e^
Wagner et al [75]; Switzerland	55 people with complicated grief; 19–68 (mean 37) years; female 93% Patients lived in Germany, Austria, and Switzerland or were native German speakers living elsewhere	Design: P + 2; outside/home Duration: 5 weeks + 3-month follow-up	Internet-based cognitive behavioral therapy for complicated grief. In *Internet-based cognitive behavioral therapy*, patients were set 2 weekly 45-minute writing assignments over a period of approximately 5 weeks, with therapist and patient communicating exclusively by email. After every second essay, patients received feedback and further instructions from the therapist. Instructions were sent within 1 working day and based on a cognitive behavioral treatment protocol but tailored to the individual patient’s needs. At the beginning of each phase of treatment, patients received psycho-education on the principles of the treatment module. *Control group: *Waiting list (received treatment 5 weeks after the treatment group termination for ethical reasons).	*Several primary outcomes: *Participants in the treatment group improved significantly relative to participants in the waiting condition on symptoms of intrusion, avoidance, maladaptive behavior, and general psychopathology, and showed a large treatment effect. Follow-up results show that this improvement was maintained after 3 months. *Research target: *Health benefits

^a ^Articles were identified in a comprehensive search in Medline, EMBASE, PsycINFO, Cochrane Central Register of Controlled Trials, and IEEE Xplore from 1990 to November 2011, and were published in the time period 2002–2011.

^b ^Main author, main reference.

^c ^Number of clinicians, number of patients with diagnosis, age range (mean) of patients, percentage female patients.

^d ^Design (P = parallel group design, CO = crossover, C = cluster, F = factorial, O = other, U = unclear; + number of study arms), where symptom reporting took place (outside or in the home; or inside a clinic), and duration of intervention.

^e ^Main findings are in general presented as in the original article and refer to primary outcome if clearly defined and secondary outcomes considered relevant for the scope of the study. Research targets refers to the six areas of health service quality defined by the Institute of Medicine [43].

### Research Targets Relevant to Electronic Symptom Reporting

According to the IOM categories of health service outcomes [43], the most common research target was disease-specific health benefits at the patient level; and, second, to provide patient-centered care (Table 4, Table 5, Table 6, and Table 7). Some of the studies also aimed at more efficient utilization of the health care system to reduce cost. The consultation support studies mainly aimed at providing patient-centered care, while monitoring and self-management studies mainly aimed for patient health benefits. The studies that aimed for reduced health care costs were all in the monitoring category, except for 1 in the self-management category.

The main research focus is presented according to the resulting health service innovations and patient groups in Multimedia Appendix 3.

## Discussion

### Principal Findings on Patient Groups, Health Service Innovations, and Research Targets

Of 642 records identified, 32 articles representing 29 studies were included. The articles were published from 2002, most (24/32, 75%) during the last 5 years. Nearly two-thirds of the articles had a female first author.

We categorized the studies into five patient groups: respiratory and lung diseases, cancer, psychiatry, cardiovascular diseases, diabetes, or a mix of these. All included studies, except 1 [66], focused on long-term conditions or issues that must take into account fluctuation in condition intensity and variations in how they influence the patient’s life.

The content analysis identified four categories of health service innovations: consultation support, monitoring with clinician support, self-management with clinician support, and therapy.

The research targets in the group of articles on consultation support were mainly patient-centered outcomes, while the articles on monitoring and self-management mainly aimed to demonstrate health benefits. The studies aiming for reduced health care costs were all in the subgroup of monitoring articles, except for 1 study on self-management*.*

### Interpretation of Results

We found that 75% of the articles were published during the last 5 years, which indicates that this is a growing field. The fact that all studies, except 2, were conducted in Western countries is not surprising, since these are the countries with the highest e-readiness [6].

It is no surprise, either, that nearly all the included studies focused on long-term conditions. In emergency or acute conditions, the time frame for decision making is short, and health professionals often need to make decisions on behalf of patients. In long-term conditions, however, the time frame is longer, and the decision process is often shared between the patient and the health care professional. Partnership or shared decision making is essential to improve the pathways for patients with long-term conditions who face complex and repeated decision making processes [9]. Supporting that decision process with self-management and patient education through technology makes sense.

Most of the consultation support innovation studies were conducted in the cancer patient group (5/7), most of the monitoring studies were in the respiratory and lung diseases (8/12), and the self-management studies were conducted mainly in psychiatry (4/9) or respiratory and lung diseases (4/9). Cancer patients who receive chemotherapy or radiation therapy (or both) for a period from 6 months to a year could theoretically benefit from both monitoring and self-management approaches, in the same way as patients with chronic obstructive pulmonary disease or asthma. Yet, electronic symptom reporting for this group of patients has mostly been studied in the context of consultation support. Likewise, electronic consultation support has not been studied in patients with chronic conditions such as chronic obstructive pulmonary disease and asthma. We can see no theoretical or practical reason to believe that these groups would not benefit in the same way as those with other long-term conditions. Thus, we are puzzled by the obvious blanks in our cross-tables of patient group by health service innovation, and that electronic symptom reporting systems seems to reflect the conventional approach in each health service field. We are concerned that health service innovations that may benefit all patients with long-term conditions are being introduced in a diagnosis-specific context. This makes it difficult for researchers and clinicians to glean more general lessons from the field. As we discuss further in the next paragraph, systems deviating from this conventional approach might benefit health care service quality. Based on this, we suggest that the field would benefit from the identification of general theoretical principles that are relevant to all electronic symptom reporting interventions, across diagnostic patient groups.

We identified four types of health service interventions, and we believe these four represent the full spectrum of services associated with electronic symptom reporting. One of the health service innovations groups, the consultation support group, was very different from the other three, while the monitoring, self-management support, and therapy groups partly overlapped. These three represent a continuum with increasing focus on treatment through electronic communication, and decreasing face-to-face or telephone contact. The ideal electronic symptom reporting service should provide both consultation support and elements of monitoring and self-management support, and when this is not enough, to support the therapeutic relationship whenever this is feasible.

The studies aiming at reduced health care costs were all in the monitoring category, except for 1 self-management study. The 4 studies defined as equivalence studies in part 2 of this review [1], which all belong to the self-management group, could be expected to focus on health care costs, but this is not the case. Equivalence here refers to the intervention hypothesis relative to the control, and not to equivalence with regard to cost. Only 1 of these studies focused on cost effectiveness, and then as its second aim [70], while the other 3 did not formally analyze cost effectiveness. However, Vernmark et al discussed cost effectiveness with regard to spent therapist time [71].

### Limitations and Strengths of the Review

This is the first review to address this emerging field and to provide a systematic overview. One of the main strengths of this review is the comprehensive search. We searched all the databases recommended by Cochrane [35] (chapter 6), in addition to the IEEE Xplore and the PsycINFO databases. All searches, except those in IEEE Xplore, included 115 or more search terms, and we adapted the searches to the individual databases. Compared with other reviews, in this review we judged a quite high percentage of the identified records (444) in our searches as relevant articles (32).

Another main strength is that the selection and data extraction strategies were based on the Cochrane recommendations.

That the review was based on the most mature research in the field, the RCT trials [32,33], is also an important strength. We are familiar with the challenges related to the use of RCTs in medical informatics [82], which therefore are not always applied to test new complex interventions. Limiting our review to RCTs might have led us to miss interventions that could be relatively mature but not tested in an RCT. Even when considering guidelines for reporting medical informatics research [83], we did not see any other way of identifying mature interventions, without simultaneously including a large body of pilot studies and feasibility trials. Evidence-based medicine with RCTs as basic methodology is widely accepted as one important facet in improving clinical practice or patient outcome [84]. However, within the constraints of the review, we did not consider studies using other methods that might have contributed to knowledge about electronic symptom reporting.

Despite using a very comprehensive search strategy, we might have lost articles in the adaptation and translation of the search strategies for the different databases. In addition, we may have missed search words, resulting in overlooked articles. When designing this review, we decided not to include articles based on hand searching of reference lists, due to the Cochrane warning [35] (Cochrane 10.2.2.3, Citation bias). Nevertheless, we checked the reference lists to get a sense of the completeness of our search. We read abstracts for all references where the title included RCT and technology implying communication, and if the abstract seemed relevant, we read the full article. We repeated this process twice for new articles we identified. Our check showed that we did not capture some psychiatry articles—mainly those that focused on a fully electronic therapeutic relationship. In these articles, the electronic symptom reporting and responses to the specific symptoms were a part of the whole picture. We acknowledge this as a blank in our review, as only 1 article from this area came up with our applied search strategy. We propose that this area merits its own review, using psychiatry terminology. We suggest not focusing so much on symptom and its synonyms as search words, since they were lacking in many of these studies, but to include specific search strings such as Interapy [85,86], Internet-based therapy [86,87], Internet-based treatment [86,88-91], and online therapeutic relationship [86].

We included the 6 psychiatry studies, as we identified them in our originally designed search strategy. If they had not been included, the self-management category would have been reduced, and been less convincing, and the focus for self-management would mainly have been on respiratory and lung diseases. On the other hand, if we had conducted a search that had covered the psychiatry field better, we hypothesize, based on the studies from the reference lists, that the psychiatry studies would have been a mix of self-management and therapy studies. However, further research will have to confirm this hypothesis.

### Future Research

The finding that nearly two-thirds of the articles had a female first author was surprising and actually something we did not look for, but was immediately obvious. This is much higher than is common in medicine, where only a few American journals have up to 30% female authors, while all others have less [92]. We do not have an explanation for this finding, but female researchers might be more engaged than their male colleagues in patient self-empowerment, defined as “a state in which an individual possesses a relatively high degree of actual power—that is, a genuine potential for making choices” [93] (p. 40). Further studies are, however, needed to investigate this observation.

As mentioned above, health service innovations in this area have so far mainly been developed and tested in the context of a given diagnosis. However, many possible ICPC-based patient groups are not represented in our findings. This is probably only a question of time, since the prereview, which was not limited to RCTs, identified 15 studies on musculoskeletal disease, 8 on gastrointestinal diseases, 8 on neurological diseases, 6 on human immunodeficiency syndrome/acquired immunodeficiency syndrome, and a large group that was not possible to categorize based on the information in the abstract [11]. These were in general pilot studies, where the next step probably is an RCT, if the innovation proves to be feasible.

What we have today is a highly heterogeneous field, where authors rarely seem to build on the experiences gained in other diagnostic settings. The fact that almost all of the patient groups had long-term conditions suggests that long-term conditions have commonalities that make this kind of intervention desirable. Future research should examine whether a common generic approach to electronic symptom reporting, regardless of diagnosis, could be useful.

A total of 3 self-management studies (4 articles) [66,67,73,74] and 3 monitoring studies [54,55,57] mainly used computer-tailored feedback to the patients. None of these studies focused on health care costs, even if these interventions may be the most promising ones to save both time and money for the health care system, compared with monitoring and self-management, where the physician or the nurse is the main communication partner. Therefore, health care costs should be an outcome in future computer-tailored feedback studies. This includes the 3 self-management studies [66,67,73,74] and the 2 monitoring studies [54,57] mentioned above, for which part 2 of this review confirms that they have acceptable methodological quality and that the hypothesis is confirmed [1].

In this regard, the Yardley et al study deserves some special attention [66]. This is the only study where no human recourses were involved on the provider side. The computer system’s tailored advice for patients with minor respiratory problems resulted in a higher level of enablement, higher satisfaction, better understanding of the illness, and a modest effect on reduced consultation rates [66] (see Part 2 of this review [1]). This concept is an excellent example of a Web-based decision support system for patients that seems to both help the patients and save time for patients, the health care system, and health care professionals. However, future studies of this concept and other Web-based decision support systems for patients, and investigation of their effect in routine care, are necessary.

In addition to the possible health cost benefits, giving the patient the opportunity to register symptoms continually and providing an interactive-feedback learning mechanism can provide the stimulus for the patient to build the necessary confidence to handle symptoms and self-management, and in this way support patient centeredness. We support Guendelman and colleagues’ suggestion that easy-to-use electronic devices “may be useful tools to empower children to provide their own care while reducing asthma symptoms and health care use in pediatric settings” [54], and might even be considered as a motivating and exciting tool for children with asthma. This idea is supported by the study of Jan et al, where several children reported that using the tool they received was fun and that it reminded them to take their medication [55]. Therefore, further research is needed to discover both the motivating (fun) and the self-management effects of technology “toys” with interactive-feedback learning mechanisms to handle symptoms.

Systematic use or reuse of electronically reported symptoms might also be useful in syndromic surveillance [94,95], to make clinicians aware of community trends and to enable them to issue the right tests and improve their diagnostic assessment [96]. There are several examples of patients reporting symptoms through the Internet for syndromic surveillance [97-100] and of relevant surveillance information being produced based on what people report on the Internet [101]. Whether symptoms reported before a consultation or reported to a decision support systems, such as that of Yardley et al [66], can be re-used for syndromic surveillance and thus result in a double effect should be investigated.

Investigating what the opportunity to easily contact care providers means to patients with long-term conditions, in terms of feeling secure, appears necessary, as nearly all of the patients who contacted the e-coach in the study of Leveille and colleagues were interested in further coaching [50,81], although the intervention had not been that promising regarding detection of screened conditions, symptom ratings, and quality of life [50].

With regard to research targets, the most interesting finding may be that none of the trials focused on safety, timeliness, and equity. Timeliness—that is, reducing delays for both providers and receivers of health care, for example, avoiding cancellation of surgery—is an area where we expect electronic symptom reporting to have a positive impact. As the mobile phone seems to narrow the digital divide [102], electronic symptom reporting might as well improve equity—that is, that health care does not vary in quality because of gender, ethnicity, geographic location, and socioeconomic status. Studies addressing these issues are needed to investigate the potential benefits.

### Implications for Practice

The recent large increase in studies being conducted in electronic symptom reporting, as also shown in our preliminary review [11], reflects the establishment of a new concept of improved patient centeredness. In addition, 88% of doctors express that they want their patients to report health indicators via mobile devices, and 40% of doctors believe it can reduce the number of office visits, according to a company that both creates products for health care companies and conducts research [103]. Accordingly, some communities and countries have already taken serious steps to achieve maximum benefits from these types of innovations. Plans to include patient-reported information as part of the electronic health record [104] have been developed. Sweden is now establishing an electronic health record and personal health record to make it possible for the patient to read health record information written by their providers, and for the health care professional to read specific parts of the patient’s personal health record describing symptoms and health information relevant to the patient’s problems and future consultations [105,106]. England’s Department of Health has just announced that they want their general practitioners to prescribe apps rather than doctors’ visits whenever possible [107-110]. They want patients to use mobile devices to monitor and track their health status, and to identify—and if possible solve—the problem before they request a visit [107-110]. They suggest these initiatives will improve quality, save money, and give patients more control over their own health. In addition, these initiatives will probably also inspire other counties to establish similar strategies. We welcome these initiatives, but recommend basing design and implementation plans on research with regard to how the technology can be used to provide safe, effective, patient-centered, timely, efficient, and equitable health care.

### Conclusion

The RCT-based research on electronic symptom reporting has developed enormously since 2002, with 75% of the articles published during the last 5 years. This indicates that a new concept to improve patient centeredness is being established. So far, the research has focused on five specific patient groups and health conditions: cancer, respiratory and lung diseases, cardiovascular disease, psychiatry, and diabetes. The evidence from RCTs can be structured into four health service innovation categories: consultation support, monitoring with clinician support, self-management with clinician support, and therapy. Most of the research (72%) has been conducted within the following four combinations: consultation support innovation in the cancer patient group (5/29, 17%), monitoring innovation in the respiratory and lung diseases patient group (8/29, 28%), and self-management innovation in the psychiatry patient group (4/29, 14%) and in the respiratory and lung diseases patient group (4/29, 14%). New patient groups, and combinations of patient groups and the four identified health service innovations, are expected in the near future. We suggest that the development of a generic (not diagnosis-specific) model for electronic patient symptom reporting for long-term conditions may benefit the development of this field.

## Multimedia Appendix 1

Medline search strategy and search terms.

## Multimedia Appendix 2

The classification process based on abstracts from the database searches.

## Multimedia Appendix 3

The main focus of the research targets presented according to health service innovations and patient groups.

## Abbreviations

ICPC: International Classification of Primary Care
IOM: Institute of Medicine
MeSH: Medical Subject Headings
PRISMA: Preferred Reporting Items for Systematic Reviews and Meta-analyses
RCT: Randomized Controlled Trial

## References

[1] Johansen MA, Berntsen GKR, Schuster T, Henriksen E, Horsch A (2012). Electronic Symptom Reporting Between Patient and Provider for Improved Health Care Service Quality: A Systematic Review of Randomized Controlled Trials. Part 2: Methodological Quality and Effects. J Med Internet Res.

[2] Nijland N, van Gemert-Pijnen JE, Boer H, Steehouder MF, Seydel ER (2009). Increasing the use of e-consultation in primary care: results of an online survey among non-users of e-consultation. Int J Med Inform.

[3] Kummervold PE, Gammon D, Bergvik S, Johnsen JA, Hasvold T, Rosenvinge JH (2002). Social support in a wired world: use of online mental health forums in Norway. Nord J Psychiatry.

[4] Webb PM, Zimet GD, Fortenberry JD, Blythe MJ (1999). Comparability of a computer-assisted versus written method for collecting health behavior information from adolescent patients. J Adolesc Health.

[5] Millsopp L, Frackleton S, Lowe D, Rogers SN (2006). A feasibility study of computer-assisted health-related quality of life data collection in patients with oral and oropharyngeal cancer. Int J Oral Maxillofac Surg.

[6] Andreassen HK, Bujnowska-Fedak MM, Chronaki CE, Dumitru RC, Pudule I, Santana S, Voss H, Wynn R (2007). European citizens' use of E-health services: a study of seven countries. BMC Public Health.

[7] Kaplan B, Brennan PF (2001). Consumer informatics supporting patients as co-producers of quality. J Am Med Inform Assoc.

[8] Randeree E, Whetstone M, Wilson EV (2009). Personal health records: patients in control. Patient-Centered E-Health.

[9] Drake RE, Deegan PE, Woltmann E, Haslett W, Drake T, Rapp CA (2010). Comprehensive electronic decision support systems. Psychiatr Serv.

[10] Drake RE, Cimpean D, Torrey WC (2009). Shared decision making in mental health: prospects for personalized medicine. Dialogues Clin Neurosci.

[11] Johansen MA, Henriksen E, Berntsen G, Horsch A (2011). Electronic symptom reporting by patients: a literature review. Stud Health Technol Inform.

[12] Sciamanna CN, Diaz J, Myne P (2002). Patient attitudes toward using computers to improve health services delivery. BMC Health Serv Res.

[13] Benoit A, Dykes P, Chang F, Gertman P, Vandever W, Li Q, Wald J (2007). Using electronic questionnaires to collect patient reported history. AMIA Annu Symp Proc.

[14] Johansen MA, Berntsen G, Shrestha N, Bellika JG, Johnsen JA (2011). An exploratory study of patient attitudes towards symptom reporting in a primary care setting. Benefits for medical consultation and syndromic surveillance?. Methods Inf Med.

[15] Buzaglo JS, Millard JL, Ridgway CG, Ross EA, Antaramian SP, Miller SM, Meropol NJ (2007). An Internet method to assess cancer patient information needs and enhance doctor-patient communication: a pilot study. J Cancer Educ.

[16] Wald JS, Businger A, Gandhi TK, Grant RW, Poon EG, Schnipper JL, Volk LA, Middleton B (2010). Implementing practice-linked pre-visit electronic journals in primary care: patient and physician use and satisfaction. J Am Med Inform Assoc.

[17] Adamson SC, Bachman JW (2010). Pilot study of providing online care in a primary care setting. Mayo Clin Proc.

[18] Kummervold PE, Trondsen M, Andreassen H, Gammon D, Hjortdahl P (2004). [Patient-physician interaction over the internet]. Tidsskr Nor Laegeforen.

[19] Head BA, Keeney C, Studts JL, Khayat M, Bumpous J, Pfeifer M (2011). Feasibility and Acceptance of a Telehealth Intervention to Promote Symptom Management during Treatment for Head and Neck Cancer. J Support Oncol.

[20] Porter SC, Kohane IS, Goldmann DA (2005). Parents as partners in obtaining the medication history. J Am Med Inform Assoc.

[21] de Jongste JC, Carraro S, Hop WC, Baraldi E, CHARISM Study Group (2009). Daily telemonitoring of exhaled nitric oxide and symptoms in the treatment of childhood asthma. Am J Respir Crit Care Med.

[22] Artinian NT, Harden JK, Kronenberg MW, Vander Wal JS, Daher E, Stephens Q, Bazzi RI (2003). Pilot study of a Web-based compliance monitoring device for patients with congestive heart failure. Heart Lung.

[23] Ross SE, Moore LA, Earnest MA, Wittevrongel L, Lin CT (2004). Providing a web-based online medical record with electronic communication capabilities to patients with congestive heart failure: randomized trial. J Med Internet Res.

[24] Gaertner J, Elsner F, Pollmann-Dahmen K, Radbruch L, Sabatowski R (2004). Electronic pain diary: a randomized crossover study. J Pain Symptom Manage.

[25] Miller DM, Moore SM, Fox RJ, Atreja A, Fu AZ, Lee JC, Saupe W, Stadtler M, Chakraborty S, Harris CM, Rudick RA (2011). Web-based self-management for patients with multiple sclerosis: a practical, randomized trial. Telemed J E Health.

[26] Lieberman DZ, Kelly TF, Douglas L, Goodwin FK (2010). A randomized comparison of online and paper mood charts for people with bipolar disorder. J Affect Disord.

[27] Ljótsson B, Hedman E, Andersson E, Hesser H, Lindfors P, Hursti T, Rydh S, Rück C, Lindefors N, Andersson G (2011). Internet-delivered exposure-based treatment vs. stress management for irritable bowel syndrome: a randomized trial. Am J Gastroenterol.

[28] Eysenbach G, Diepgen TL (1998). Epidemiological data can be gathered with world wide web. BMJ.

[29] Barnason S, Zimmerman L, Nieveen J, Schulz P, Miller C, Hertzog M, Tu C (2009). Influence of a symptom management telehealth intervention on older adults' early recovery outcomes after coronary artery bypass surgery. Heart Lung.

[30] Zimmerman L, Barnason S, Hertzog M, Young L, Nieveen J, Schulz P, Tu C (2011). Gender differences in recovery outcomes after an early recovery symptom management intervention. Heart Lung.

[31] Lorenzi NM, Riley RT, Blyth AJ, Southon G, Dixon BJ (1997). Antecedents of the people and organizational aspects of medical informatics: review of the literature. J Am Med Inform Assoc.

[32] Campbell NC, Murray E, Darbyshire J, Emery J, Farmer A, Griffiths F, Guthrie B, Lester H, Wilson P, Kinmonth AL (2007). Designing and evaluating complex interventions to improve health care. BMJ.

[33] Campbell M, Fitzpatrick R, Haines A, Kinmonth AL, Sandercock P, Spiegelhalter D, Tyrer P (2000). Framework for design and evaluation of complex interventions to improve health. BMJ.

[34] Moher D, Liberati A, Tetzlaff J, Altman DG, PRISMA Group (2009). Preferred reporting items for systematic reviews and meta-analyses: the PRISMA Statement. Open Med.

[35] Higgins JPT, Green S (2011). Cochrane Handbook for Systematic Reviews of Interventions, Version 5.1.0 [updated March 2011].

[36] Eklund AM (2011). Medical knowledge evolution query constraining aspects. Stud Health Technol Inform.

[37] Wong SS, Wilczynski NL, Haynes RB (2006). Comparison of top-performing search strategies for detecting clinically sound treatment studies and systematic reviews in MEDLINE and EMBASE. J Med Libr Assoc.

[38] Johansen MA (2012). The Norwegian Centre for Integrated Care and Telemedicine.

[39] Evans D, Pearson A (2001). Systematic reviews of qualitative research. Clin Eff Nurs.

[40] Devers KJ (2011). Qualitative methods in health services and management research: pockets of excellence and progress, but still a long way to go. Med Care Res Rev.

[41] Pope C, Ziebland S, Mays N (2000). Qualitative research in health care. Analysing qualitative data. BMJ.

[42] KITH (2004). Norwegian Centre for Informatics in Health and Social Care in cooperation with "Norsk selskap for allemennmedisin".

[43] Committee on Quality of Health Care in America, Institute of Medicine (2001). Crossing the Quality Chasm: A New Health System for the 21st Century.

[44] Berry DL, Blumenstein BA, Halpenny B, Wolpin S, Fann JR, Austin-Seymour M, Bush N, Karras BT, Lober WB, McCorkle R (2011). Enhancing patient-provider communication with the electronic self-report assessment for cancer: a randomized trial. J Clin Oncol.

[45] Boyes A, Newell S, Girgis A, McElduff P, Sanson-Fisher R (2006). Does routine assessment and real-time feedback improve cancer patients' psychosocial well-being?. Eur J Cancer Care (Engl).

[46] Ruland CM, White T, Stevens M, Fanciullo G, Khilani SM (2003). Effects of a computerized system to support shared decision making in symptom management of cancer patients: preliminary results. J Am Med Inform Assoc.

[47] Ruland CM, Holte HH, Røislien J, Heaven C, Hamilton GA, Kristiansen J, Sandbaek H, Kvaløy SO, Hasund L, Ellison MC (2010). Effects of a computer-supported interactive tailored patient assessment tool on patient care, symptom distress, and patients' need for symptom management support: a randomized clinical trial. J Am Med Inform Assoc.

[48] Velikova G, Booth L, Smith AB, Brown PM, Lynch P, Brown JM, Selby PJ (2004). Measuring quality of life in routine oncology practice improves communication and patient well-being: a randomized controlled trial. J Clin Oncol.

[49] Stevens J, Kelleher KJ, Gardner W, Chisolm D, McGeehan J, Pajer K, Buchanan L (2008). Trial of computerized screening for adolescent behavioral concerns. Pediatrics.

[50] Leveille SG, Huang A, Tsai SB, Allen M, Weingart SN, Iezzoni LI (2009). Health coaching via an internet portal for primary care patients with chronic conditions: a randomized controlled trial. Med Care.

[51] Kearney N, McCann L, Norrie J, Taylor L, Gray P, McGee-Lennon M, Sage M, Miller M, Maguire R (2009). Evaluation of a mobile phone-based, advanced symptom management system (ASyMS) in the management of chemotherapy-related toxicity. Support Care Cancer.

[52] Chan DS, Callahan CW, Sheets SJ, Moreno CN, Malone FJ (2003). An Internet-based store-and-forward video home telehealth system for improving asthma outcomes in children. Am J Health Syst Pharm.

[53] Chan DS, Callahan CW, Hatch-Pigott VB, Lawless A, Proffitt HL, Manning NE, Schweikert M, Malone FJ (2007). Internet-based home monitoring and education of children with asthma is comparable to ideal office-based care: results of a 1-year asthma in-home monitoring trial. Pediatrics.

[54] Guendelman S, Meade K, Benson M, Chen YQ, Samuels S (2002). Improving asthma outcomes and self-management behaviors of inner-city children: a randomized trial of the Health Buddy interactive device and an asthma diary. Arch Pediatr Adolesc Med.

[55] Jan RL, Wang JY, Huang MC, Tseng SM, Su HJ, Liu LF (2007). An internet-based interactive telemonitoring system for improving childhood asthma outcomes in Taiwan. Telemed J E Health.

[56] Prabhakaran L, Chee WY, Chua KC, Abisheganaden J, Wong WM (2010). The use of text messaging to improve asthma control: a pilot study using the mobile phone short messaging service (SMS). J Telemed Telecare.

[57] Rasmussen LM, Phanareth K, Nolte H, Backer V (2005). Internet-based monitoring of asthma: a long-term, randomized clinical study of 300 asthmatic subjects. J Allergy Clin Immunol.

[58] Willems DC, Joore MA, Hendriks JJ, Nieman FH, Severens JL, Wouters EF (2008). The effectiveness of nurse-led telemonitoring of asthma: results of a randomized controlled trial. J Eval Clin Pract.

[59] Lewis KE, Annandale JA, Warm DL, Rees SE, Hurlin C, Blyth H, Syed Y, Lewis L (2010). Does home telemonitoring after pulmonary rehabilitation reduce healthcare use in optimized COPD? A pilot randomized trial. COPD.

[60] Lewis KE, Annandale JA, Warm DL, Hurlin C, Lewis MJ, Lewis L (2010). Home telemonitoring and quality of life in stable, optimised chronic obstructive pulmonary disease. J Telemed Telecare.

[61] Nguyen HQ, Gill DP, Wolpin S, Steele BG, Benditt JO (2009). Pilot study of a cell phone-based exercise persistence intervention post-rehabilitation for COPD. Int J Chron Obstruct Pulmon Dis.

[62] Carrasco MP, Salvador CH, Sagredo PG, Márquez-Montes J, González de Mingo MA, Fragua JA, Rodríguez MC, García-Olmos LM, García-López F, Carrero AM, Monteagudo JL (2008). Impact of patient-general practitioner short-messages-based interaction on the control of hypertension in a follow-up service for low-to-medium risk hypertensive patients: a randomized controlled trial. IEEE Trans Inf Technol Biomed.

[63] Santamore WP, Homko CJ, Kashem A, McConnell TR, Bove AA (2007). Using a telemedicine system to decrease cardiovascular disease risk in an underserved population: design, use, and interim results. Conf Proc IEEE Eng Med Biol Soc.

[64] Schwarz KA, Mion LC, Hudock D, Litman G (2008). Telemonitoring of heart failure patients and their caregivers: a pilot randomized controlled trial. Prog Cardiovasc Nurs.

[65] DeVito Dabbs A, Dew MA, Myers B, Begey A, Hawkins R, Ren D, Dunbar-Jacob J, Oconnell E, McCurry KR (2009). Evaluation of a hand-held, computer-based intervention to promote early self-care behaviors after lung transplant. Clin Transplant.

[66] Yardley L, Joseph J, Michie S, Weal M, Wills G, Little P (2010). Evaluation of a Web-based intervention providing tailored advice for self-management of minor respiratory symptoms: exploratory randomized controlled trial. J Med Internet Res.

[67] van der Meer V, Bakker MJ, van den Hout WB, Rabe KF, Sterk PJ, Kievit J, Assendelft WJ, Sont JK, SMASHING (Self-Management in Asthma Supported by Hospitals&sbquo; ICT&sbquo; Nurses and General Practitioners) Study Group (2009). Internet-based self-management plus education compared with usual care in asthma: a randomized trial. Ann Intern Med.

[68] Nguyen HQ, Donesky-Cuenco D, Wolpin S, Reinke LF, Benditt JO, Paul SM, Carrieri-Kohlman V (2008). Randomized controlled trial of an internet-based versus face-to-face dyspnea self-management program for patients with chronic obstructive pulmonary disease: pilot study. J Med Internet Res.

[69] Berger T, Caspar F, Richardson R, Kneubühler B, Sutter D, Andersson G (2011). Internet-based treatment of social phobia: a randomized controlled trial comparing unguided with two types of guided self-help. Behav Res Ther.

[70] Bergström J, Andersson G, Ljótsson B, Rück C, Andréewitch S, Karlsson A, Carlbring P, Andersson E, Lindefors N (2010). Internet-versus group-administered cognitive behaviour therapy for panic disorder in a psychiatric setting: a randomised trial. BMC Psychiatry.

[71] Vernmark K, Lenndin J, Bjärehed J, Carlsson M, Karlsson J, Oberg J, Carlbring P, Eriksson T, Andersson G (2010). Internet administered guided self-help versus individualized e-mail therapy: A randomized trial of two versions of CBT for major depression. Behav Res Ther.

[72] Oerlemans S, van Cranenburgh O, Herremans PJ, Spreeuwenberg P, van Dulmen S (2011). Intervening on cognitions and behavior in irritable bowel syndrome: A feasibility trial using PDAs. J Psychosom Res.

[73] Glasgow RE, Nutting PA, King DK, Nelson CC, Cutter G, Gaglio B, Rahm AK, Whitesides H (2005). Randomized effectiveness trial of a computer-assisted intervention to improve diabetes care. Diabetes Care.

[74] Williams GC, Lynch M, Glasgow RE (2007). Computer-assisted intervention improves patient-centered diabetes care by increasing autonomy support. Health Psychol.

[75] Wagner B, Knaevelsrud C, Maercker A (2006). Internet-based cognitive-behavioral therapy for complicated grief: a randomized controlled trial. Death Stud.

[76] Lorig KR, Holman H (2003). Self-management education: history, definition, outcomes, and mechanisms. Ann Behav Med.

[77] Wagner EH, Austin BT, Davis C, Hindmarsh M, Schaefer J, Bonomi A (2001). Improving chronic illness care: translating evidence into action. Health Aff (Millwood).

[78] Wolpin S, Berry D, Austin-Seymour M, Bush N, Fann JR, Halpenny B, Lober WB, McCorkle R (2008). Acceptability of an Electronic Self-Report Assessment Program for patients with cancer. Comput Inform Nurs.

[79] McCann L, Maguire R, Miller M, Kearney N (2009). Patients' perceptions and experiences of using a mobile phone-based advanced symptom management system (ASyMS) to monitor and manage chemotherapy related toxicity. Eur J Cancer Care (Engl).

[80] Willems DC, Joore MA, Hendriks JJ, Wouters EF, Severens JL (2007). Cost-effectiveness of a nurse-led telemonitoring intervention based on peak expiratory flow measurements in asthmatics: results of a randomised controlled trial. Cost Eff Resour Alloc.

[81] Allen M, Iezzoni LI, Huang A, Huang L, Leveille SG (2008). Improving patient-clinician communication about chronic conditions: description of an internet-based nurse E-coach intervention. Nurs Res.

[82] Liu JL, Wyatt JC (2011). The case for randomized controlled trials to assess the impact of clinical information systems. J Am Med Inform Assoc.

[83] Talmon J, Ammenwerth E, Brender J, de Keizer N, Nykänen P, Rigby M (2009). STARE-HI--Statement on reporting of evaluation studies in Health Informatics. Int J Med Inform.

[84] Sackett DL, Haynes RB, Tugwell P (1985). Clinical Epidemiology: A Basic Science for Clinical Medicine. 1st edition.

[85] Lange A, Rietdijk D, Hudcovicova M, van de Ven JP, Schrieken B, Emmelkamp PM (2003). Interapy: a controlled randomized trial of the standardized treatment of posttraumatic stress through the internet. J Consult Clin Psychol.

[86] Knaevelsrud C, Maercker A (2007). Internet-based treatment for PTSD reduces distress and facilitates the development of a strong therapeutic alliance: a randomized controlled clinical trial. BMC Psychiatry.

[87] Andersson G, Bergström J, Holländare F, Carlbring P, Kaldo V, Ekselius L (2005). Internet-based self-help for depression: randomised controlled trial. Br J Psychiatry.

[88] Berger T, Hohl E, Caspar F (2009). Internet-based treatment for social phobia: a randomized controlled trial. J Clin Psychol.

[89] Perini S, Titov N, Andrews G (2009). Clinician-assisted Internet-based treatment is effective for depression: randomized controlled trial. Aust N Z J Psychiatry.

[90] Titov N, Andrews G, Choi I, Schwencke G, Mahoney A (2008). Shyness 3: randomized controlled trial of guided versus unguided Internet-based CBT for social phobia. Aust N Z J Psychiatry.

[91] Wims E, Titov N, Andrews G, Choi I (2010). Clinician-assisted Internet-based treatment is effective for panic: A randomized controlled trial. Aust N Z J Psychiatry.

[92] Baethge C (2008). First authors in Deutsches Arzteblatt: women are catching up. The number of female authors in medical literature is increasing, but is still considerably lower than that of male authors and corresponds to the proportion of women working in academic medicine. Dtsch Arztebl Int.

[93] Tones K, Tilford S (2001). Health Promotion: Effectiveness, Efficiency and Equity. 3rd edition.

[94] Mandl KD, Overhage JM, Wagner MM, Lober WB, Sebastiani P, Mostashari F, Pavlin JA, Gesteland PH, Treadwell T, Koski E, Hutwagner L, Buckeridge DL, Aller RD, Grannis S (2004). Implementing syndromic surveillance: a practical guide informed by the early experience. J Am Med Inform Assoc.

[95] Reingold A (2003). If syndromic surveillance is the answer, what is the question?. Biosecur Bioterror.

[96] Foldy SL (2004). Linking better surveillance to better outcomes. MMWR Morb Mortal Wkly Rep.

[97] Friesema IH, Koppeschaar CE, Donker GA, Dijkstra F, van Noort SP, Smallenburg R, van der Hoek W, van der Sande MA (2009). Internet-based monitoring of influenza-like illness in the general population: experience of five influenza seasons in The Netherlands. Vaccine.

[98] Marquet RL, Bartelds AI, van Noort SP, Koppeschaar CE, Paget J, Schellevis FG, van der Zee J (2006). Internet-based monitoring of influenza-like illness (ILI) in the general population of the Netherlands during the 2003-2004 influenza season. BMC Public Health.

[99] van Noort SP, Muehlen M, Rebelo de Andrade H, Koppeschaar C, Lima Lourenço JM, Gomes MG (2007). Gripenet: an internet-based system to monitor influenza-like illness uniformly across Europe. Euro Surveill.

[100] Eysenbach G (2009). Infodemiology and infoveillance: framework for an emerging set of public health informatics methods to analyze search, communication and publication behavior on the Internet. J Med Internet Res.

[101] Eysenbach G (2011). Infodemiology and infoveillance tracking online health information and cyberbehavior for public health. Am J Prev Med.

[102] Gallaga OL The Statesman.

[103] Murphy S (2012). Mashable, Inc.

[104] Glasgow RE, Kaplan RM, Ockene JK, Fisher EB, Emmons KM (2012). Patient-reported measures of psychosocial issues and health behavior should be added to electronic health records. Health Aff (Millwood).

[105] Agerberg M (2012). Läkartidningen.

[106] Ekendahl M (2012). Inera AB.

[107] Hammond N (2012). Marie Claire.

[108] Kee E (2012). ÜberGizmo.

[109] Roberts M (2012). BBC News Health.

[110] Husain I iMedicalApps.

