# Supplementary material for: Electronic Symptom Reporting Between Patient and Provider for Improved Health Care Service Quality: A Systematic Review of Randomized Controlled Trials. Part 1: State of the Art
Source: J Med Internet Res. 2012 Oct 3;14(5):e118. doi: 10.2196/jmir.2214 (PMC3510721; doi:10.2196/jmir.2214)
Supplement: Supplementary file 1 [file jmir_v14i5e118_app1.pdf]

The MEDLINE search strategy and search terms

The search is built up around four search files (What, Who, Why, How), with a logical OR within the files, and an AND between the files. The search is based on MeSH terms and the Text Word (TW) field to search titles and abstract information.

First follow the How file (searches 1-38), then the What file (searches 40-61), then the Who file (searches 63-80), and finally the Why file (searches 82-132).

1. e-diary.tw.
2. digital media.tw.
3. technologic*.tw.
4. web.tw.
5. computer*.tw.
6. kiosk*.tw.
7. electronic*.tw.
8. internet*.tw.
9. communication* media.tw.
10. on-line communication*.tw.
11. online communication*.tw.
12. offline system*.tw.
13. off-line system*.tw.
14. offline communicat*.tw.
15. off-line communicat*.tw.
16. online system*.tw.
17. on-line system*.tw.
18. Electronic* Mail*.tw.
19. mobile phone*.tw.
20. mobile device*.tw.
21. wireless device*.tw.
22. wireless phone*.tw.
23. cell* phone*.tw.
24. hand held device*.tw.
25. telemedicine.tw.
26. store-and forward*.tw.
27. Medical Informatics/
28. technology*.tw.
29. computers/ or computers, handheld/
30. Internet/
31. Computer Terminals/ or Computer Systems/
32. Communications Media/

33. Online Systems/

34. Electronic Mail/

35. Cellular Phone/

36. Telecommunications/

37. Telemedicine/

38. Technology/

39. 1 or 2 or 3 or 4 or 5 or 6 or 7 or 8 or 9 or 10 or 11 or 12 or 13 or 14 or 15 or 16 or 17 or 18 or 19 or 20 or 21 or 22 or 23 or 24 or 25 or 26 or 27 or 28 or 29 or 30 or 31 or 32 or 33 or 34 or 35 or 36 or 37 or 38

40. symptom.tw.

41. symptoms.tw.

42. Medical History Taking/

43. medical documentation*.tw.

44. health information*.tw.

45. health history.tw.

46. health data.tw.

47. health findings.tw.

48. medical information.tw.

49. health condition*.tw.

50. medication history.tw.

51. patient-specific data.tw.

52. information about patient* preference*.tw.

53. patient preference*.tw.

54. patients' experience*.tw.

55. patient experience*.tw.

56. Health Status/

57. pain-related variable*.tw.

58. pain-response*.tw.

59. health-related variable*.tw.

60. health-response*.tw.

61. Data Collection/is, mt, td, ut [Instrumentation, Methods, Trends, Utilization]

62. 40 or 41 or 42 or 43 or 44 or 45 or 46 or 47 or 48 or 49 or 50 or 51 or 52 or 53 or 54 or 55 or 56 or 57 or 58 or 59 or 60 or 61

63. patient*.tw.

64. Patients/

65. Nurse-Patient Relations/

66. Patient Participation/

67. Patient Satisfaction/

68. Hospital-Patient Relations/

69. Patient-Centered Care/

70. Patient Access to Records/

71. Professional-Patient Relations/

72. Physician-Patient Relations/

73. Patient Preference/

74. Parents/

75. parent*.tw.

76. Patient Care/mt, td [Methods, Trends]

77. Consumer Health Information/

78. Consumer Participation/

79. Consumer Satisfaction/

80. Patient Access to Records/

81. 63 or 64 or 65 or 66 or 67 or 68 or 69 or 70 or 71 or 72 or 73 or 74 or 75 or 76 or 77 or 78 or 79 or 80

82. Self Disclosure/

83. self disclosur*.tw.

84. pre-report*.tw.

85. prereport*.tw.

86. self-report*.tw.

87. selfreport*.tw.

88. report* by patient*.tw.

89. communicat* by patient*.tw.

90. report* by parent*.tw.

91. communicat* by parent*.tw.

92. patient* report*.tw.

93. patient* communicat*.tw.

94. parent* report*.tw.

95. parent* communicat*.tw.

96. symptom* registrat*.tw.

97. symptom* report*.tw.

98. pre consultation*.tw.

99. preconsultation*.tw.

100. symptom* management*.tw.

101. symptom* assessment*.tw.

102. patient* centered information management.tw.

103. patient* driven decision support*.tw.

104. real time care.tw.

105. patient-clinician shared electronic health record*.tw.

106. prior to the consultation*.tw.

107. preference based care.tw.
108. assessment* of symptom*.tw.
109. patient-centered outcome assessment*.tw.
110. communicat* symptom* to provider*.tw.
111. report* to their clinician*.tw.
112. preference achievement.tw.
113. experience sampling.tw.
114. client* report*.tw.
115. report* by client*.tw.
116. communicat* by client*.tw.
117. health records, personal/
118. Self Care/is, mt, td [Instrumentation, Methods, Trends]
119. self-care.tw.
120. self-management*.tw.
121. tele-monitoring.tw.
122. telemonitoring.tw.
123. tele-management.tw.
124. telemanagement.tw.
125. self-testing.tw.
126. selftesting.tw.
127. selfmonitor*.tw.
128. self-monitor*.tw.
129. shared management.tw.
130. self-rated.tw.
131. selfrated.tw.
132. momentary data gathering.tw.
133. 82 or 83 or 84 or 85 or 86 or 87 or 88 or 89 or 90 or 91 or 92 or 93 or 94 or 95 or 96 or 97 or 98 or 99 or 100 or 101 or 102 or 103 or 104 or 105 or 106 or 107 or 108 or 109 or 110 or 111 or 112 or 113 or 114 or 115 or 116 or 117 or 118 or 119 or 120 or 121 or 122 or 123 or 124 or 125 or 126 or 127 or 128 or 129 or 130 or 131 or 132
134. 39 and 62 and 81 and 133
135. limit 134 to (english language and humans and yr="1990 -Current")
136. limit 135 to randomized controlled trial
