# Supplementary material for: Electronic Symptom Reporting Between Patient and Provider for Improved Health Care Service Quality: A Systematic Review of Randomized Controlled Trials. Part 1: State of the Art
Source: J Med Internet Res. 2012 Oct 3;14(5):e118. doi: 10.2196/jmir.2214 (PMC3510721; doi:10.2196/jmir.2214)
Supplement: Supplementary file 2 [file jmir_v14i5e118_app2.pdf]

Abstracts identified through MEDLINE, Embase, PsycINFO and Cochrane search

**Cochrane, PsycINFO Embase, MEDLINE**	**Not relevant**	**Potentially relevant**	**Total**
**Discussed**	33	10	43
**Agreed**	184	52	236
**Total**	217	62	279

Abstracts identified through the IEEE Xplore search

**IEEE Xplore**	**Not relevant**	**Potentially relevant**	**Total**
**Discussed**	5	1	6
**Agreed**	151	7	158
**Total**	156	8	164
