# Supplementary material for: Electronic Symptom Reporting Between Patient and Provider for Improved Health Care Service Quality: A Systematic Review of Randomized Controlled Trials. Part 1: State of the Art
Source: J Med Internet Res. 2012 Oct 3;14(5):e118. doi: 10.2196/jmir.2214 (PMC3510721; doi:10.2196/jmir.2214)
Supplement: Supplementary file 3 [file jmir_v14i5e118_app3.pdf]

Main focus of the research with references included in this review of RCTs on electronic symptom reporting, presented by health service innovation category and patient group. Patient groups are categorized by use of ICPC, plus adding Cancer as a separate group. Respiratory/lung diseases are split in three subgroups: Asthma, COPD and other. The “Mix” row includes a mix of several ICPC categories. The articles were identified in a comprehensive search in MEDLINE, Embase, PsycINFO, Cochrane Central Register of Controlled Trials and IEEE Xplore from 1990 to November 2011. All the articles were published in the time period 2002-2011. References with “and” between them are articles that belong to the same study.

**Patient groups**	**7 Consultation support studies**	**12 Monitoring with clinical support studies**	**9 Self-management with clinical support studies**	**1 Therapy study**
**Cancer**	**5 studies:**<br>More symptoms identified and discussed during consultation: [44-48]	**1 study:**<br>Morbidity of chemotherapy-related symptoms [51]		
**Respiratory / lung diseases: *Asthma***		**6 studies / 7 articles:**<br>Improved asthma outcome (symptoms and/or QoL):<br>Children: [52, 53], [54], [55]<br>Adults: [56], [57]<br>Both: [58]	**1 study:**<br>Improved asthma-related QoL [67]	
**Respiratory / lung diseases: *COPD*[a]**		**2 studies / 3 articles:**<br>-Reduced healthcare use (hospital admission, ED-visits) [59] and improved QoL [60]<br>-Feasibility of a cell phone-based exercise intervention [61]	**1 study:**<br>Reducing dyspnea with activities of daily living [68]	
**Respiratory / lung diseases: *Other***			**2 studies:**<br>Maximize transplant-related health outcomes [65]<br>Self-care in management of minor respiratory symptoms [66]	
**Cardio-vascular**		**3 studies :**<br>-Improved control of hypertension [62]<br>-Reduced cardio vascular disease risk through control of symptom, sign, activity and smoking [63]<br>-Reduced hospital readmission, Emergency Department-visits, and cost [64]		

**Psychiatry**	**1 study:**<br>More symptoms identified and discussed during consultation [49]		**4 studies:**<br>Symptom reduction for:<br>1) Social phobia [69]<br>2) Depression [71]<br><br>More effective cognitive behaviour therapy for patients with:<br>1) Panic disorder [70]<br>2) Irritable bowel syndrome [72]	**1 study:**<br>More effective cognitive-behavioural therapy for bereaved people suffering complicated grief [75]
**Diabetes**			**1 study / 2 articles:**<br>Improved quality on recommended primary care [73] and effects on outcomes<br>[74]	
**Mix**	1 study:<br>More symptoms identified and discussed during consultation [50]			
